# Proteomics and Drug Repurposing in CLL towards Precision Medicine

**DOI:** 10.3390/cancers13143391

**Published:** 2021-07-06

**Authors:** Dimitra Mavridou, Konstantina Psatha, Michalis Aivaliotis

**Affiliations:** 1 Laboratory of Biochemistry, School of Medicine, Faculty of Health Sciences, Aristotle University of Thessaloniki, GR-54124 Thessaloniki, Greece; dimitramd@auth.gr; 2 Functional Proteomics and Systems Biology (FunPATh)—Center for Interdisciplinary Research and Innovation (CIRI-AUTH), GR-57001 Thessaloniki, Greece; 3 Basic and Translational Research Unit, Special Unit for Biomedical Research and Education, School of Medicine, Aristotle University of Thessaloniki, GR-54124 Thessaloniki, Greece; 4 Institute of Molecular Biology and Biotechnology, Foundation of Research and Technology, GR-70013 Heraklion, Greece

**Keywords:** CLL, proteomics, drug repurposing, precision medicine, malignancy

## Abstract

**Simple Summary:**

Despite continued efforts, the current status of knowledge in CLL molecular pathobiology, diagnosis, prognosis and treatment remains elusive and imprecise. Proteomics approaches combined with advanced bioinformatics and drug repurposing promise to shed light on the complex proteome heterogeneity of CLL patients and mitigate, improve, or even eliminate the knowledge stagnation. In relation to this concept, this review presents a brief overview of all the available proteomics and drug repurposing studies in CLL and suggests the way such studies can be exploited to find effective therapeutic options combined with drug repurposing strategies to adopt and accost a more “precision medicine” spectrum.

**Abstract:**

CLL is a hematological malignancy considered as the most frequent lymphoproliferative disease in the western world. It is characterized by high molecular heterogeneity and despite the available therapeutic options, there are many patient subgroups showing the insufficient effectiveness of disease treatment. The challenge is to investigate the individual molecular characteristics and heterogeneity of these patients. Proteomics analysis is a powerful approach that monitors the constant state of flux operators of genetic information and can unravel the proteome heterogeneity and rewiring into protein pathways in CLL patients. This review essences all the available proteomics studies in CLL and suggests the way these studies can be exploited to find effective therapeutic options combined with drug repurposing approaches. Drug repurposing utilizes all the existing knowledge of the safety and efficacy of FDA-approved or investigational drugs and anticipates drug alignment to crucial CLL therapeutic targets, leading to a better disease outcome. The drug repurposing studies in CLL are also discussed in this review. The next goal involves the integration of proteomics-based drug repurposing in precision medicine, as well as the application of this procedure into clinical practice to predict the most appropriate drugs combination that could ensure therapy and the long-term survival of each CLL patient.

## 1. Introduction

### 1.1. Currently Known Pathophysiology, Molecular Diagnosis and Treatment Strategies in CLL

**Figure 1:**
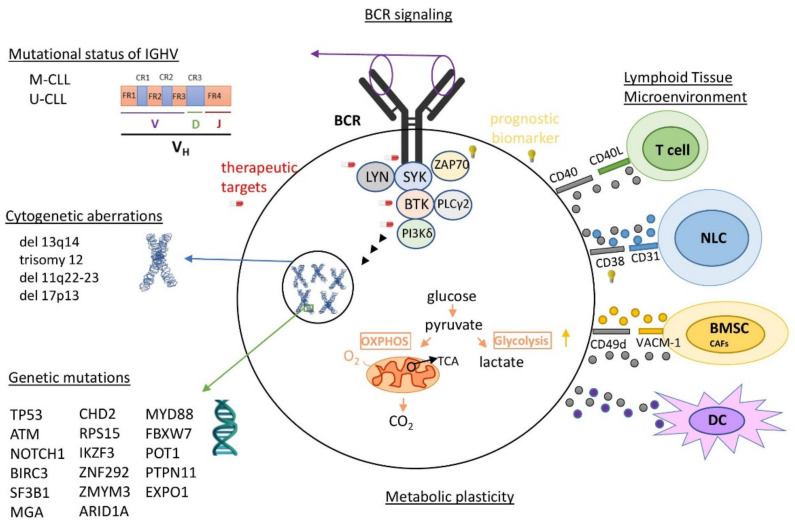
Currently known heterogeneity in the pathophysiology of CLL. Some of the most important elements of CLL pathophysiology are: (1) highly varying genomic mutations, (2) loss or addition of large amounts of chromosomal material, (3) mutational status of variable region of IGHV, (4) frequent activation of BCR signaling and (5) continuous proliferating signals from the cancerous microenvironment. IGHV: immunoglobulin heavy chain variable region genes; M-CLL: mutated CLL; U-CLL: unmutated CLL; BCR: B cell receptor; NLC: nurse-like cells; BMSC: bone marrow stromal cells; DC: dendritic cells; OXPHOS: oxidative phosphorylation; TCA: citric acid cycle.

CLL is the most frequent lymphoproliferative disease in the western world [1,2] characterized by the clonal proliferation and progressive accumulation of mature, typically CD5-positive B-cells in the blood, bone marrow, and secondary lymphoid tissues [2,3,4]. It shows a high biological, genetical, molecular and clinical diversity [1,5], projecting its highly heterogenous pathophysiology (Figure 1, Table S1). Among the known features with clinical relevance in the pathobiology of CLL are the highly genetic mutations acting either independently or in combination with chromosomal rearrangements [1,5]. Driver mutations have been associated with adverse clinical outcomes, and thus serve as biomarkers, indicators of therapeutic options or as potential therapeutic targets (Table S1) [2,4,5,6,7,8]. Somatic mutations in immunoglobulin heavy chain variable region gene (IGHV), activating B cell receptor (BCR)-signaling kinases lead to the lower survival and proliferation of CLL cells, providing patients with “mutated” M-CLL, which is a better clinical outcome vs. “unmutated” U-CLL patients [2,9]. It is important to mention that the signaling of UM-CLL is generally highly responsive to the antigenic stimulus, while M-CLL are anergic. Continual or repetitive BCR signaling adds further complexity in CLL pathogenesis, contributing to autophagy regulation, promoting tumor survival, proliferation, and consequently tumor progression [10]. Complex karyotype (CK), defined by the presence of at least three genetic abnormalities in the same clone, is detectable in 14–34% of CLL cases and it is recommended as a new negative prognostic biomarker associated with an adverse outcome and worse response to chemoimmunotherapy [11,12,13,14,15]. Other intriguing features of vital significance in the growth, survival, and drug resistance of CLL cells are metabolic plasticity and signals from the lymphoid tissue microenvironment (LTME) [2,4]. Metabolic plasticity involves the main metabolic pathways of mitochondrial biogenesis and bioenergetics, ROS production, and adaptation to intrinsic oxidative stress, found to be elevated in CLL [16]. LTME produces various essential proteins and metabolites [3,17] modulating the redox and metabolic state of CLL cells [18] and switching either to oxidative phosphorylation (OXPHOS) or glycolysis [19]. Furthermore, enhanced BCR signaling induces the metabolic activation of CLL cells through OXPHOS, energetically supporting the transcription and translation processes [20].

The molecular diagnostic criteria in CLL guidelines and beyond traditional Rai or Binet staging [21] include (i) the co-expression of CD5 with the B-cell antigens CD19 and CD20, (ii) characteristically lower levels of surface immunoglobulin, CD20, and CD79b (vs. normal B cells), (iii) the expression of kappa or lambda immunoglobulin (Ig) light chains [3,22] and (iv) the identification of specific gene mutations and serum markers [2,3,22,23]. Additionally, the CLL International Prognostic Index (CLL-IPI) proposes a weighted grading of five parameters: (i) *TP53* dysfunction, (ii) mutational status of IGHV, (iii) serum level of β2-microglobulin, (iv) clinical stage, and (v) age [3]. Furthermore, an increasing number of studies are supporting the use of new biomarkers for the diagnosis, prognosis of clinical course and therapeutic decision, such as newly approved driver genes [5,24,25,26,27,28,29,30] serum micro-RNAs [31], etc. Interestingly, assessment of the minimal residual disease (MRD), referring to the small numbers of CLL cells that remain in patients in remission during or after treatment, is an emerging prognostic biomarker of progression-free and overall survival [23,32].

A plethora of pharmacological targets have been investigated in CLL (Table S2, Figure S1). Patients, according to their clinical history, are prioritized to therapeutic options, including chemotherapy, immunotherapy (IT), chimeric antigen receptor and other targeted therapeutic strategies, used alone or in combination. More specifically, chemotherapeutic agents are still used in many cases as a first-line treatment. [2,3]. In chemo-immunotherapy (CIT), mAbs bind in the surface antigens of CLL cells, resulting in apoptosis, complement-dependent cytotoxicity (CDC) or antibody-dependent cellular cytotoxicity (ADCC). Different combinations of several agents have been reported and evaluated in several publications [2,3,22,33,34]. Inhibitors targeting the aberrantly regulated components of apoptosis, and of BCR signaling in CLL [2,3,22,33,35,36], have started to replace CIT, in first- and second-line indications [3,36] and many other new generations or under investigation agents [36,37,38,39]. The therapy using CAR-T cells represents a recent therapeutic option for some CLL patients [40,41]. Finally, among the promising CLL therapies under investigation targeting several deregulated pathways are the cross-talk between CLL cells, the tumor microenvironment [42,43], the Wnt signaling pathway [44], various miRNAs [45], the Notch2 signaling pathway [46], the mitochondrial metabolism [16] and the epigenetic modifications [47].

### 1.2. The Knowledge Gap in the Fight against CLL

There is still a translational gap between basic knowledge and clinical application in CLL. Despite current therapeutic strategies and improvements, there are an increasing number of deaths in accordance with the increasing incidence rates and the second primary malignancies (SPMs) [48,49,50,51,52,53]. Unknown genetic risk factors related to specific SMPs in patients, individual complex karyotypes, genetic mutations, altered signaling pathways, individual tumor microenvironments, recurrent expanded or diminished genetic alterations and “ad hoc” therapies, and drugs combinations without restrictive guideline based on characteristic biomarkers, are among some of the reasons [3,5,8,36,54]. The consequences are inadequate drug response, MRD and drug resistance [5,32,36,54]. To deal with the missing information regarding the molecular etiology, complexity, and heterogeneity of the disease traits, it is important to decode in detail the different molecular elements and their intricate interplay driving CLL phenotypes, to allow the selection of more effective and safe treatment options, as well as long-term remissions.

### 1.3. Proteomics and Drug Repurposing in the Fight against CLL

The springboard to a more precise and holistic molecular perspective of the pathobiology of CLL patients is through the contribution of omics and systems biology approaches that enable improved early and accurate diagnosis, prognosis, and therapeutic insights. The identification and validation of more specific signatures and drug targets elucidating the underlying mechanism of action, as well as the application of an individualized, well-tolerated, and safe therapeutic protocol, could ensure the long-term, good-quality survival of CLL patients [5,8]. Exploiting omics results, or high quality and well-documented omics data available in public repositories can be used for a comprehensive biological insight in CLL pathobiology. The meta- and re-analysis of such omics data can unravel characteristic differences responsible for the deregulation of important molecular networks and pathways in CLL. It is imperative that these differences are scrupulously investigated for their unique essentiality in different CLL phenotypes, categorizing patients into further subgroups, and identifying specific druggable targets for the selection of a more precise treatment. Furthermore, since the rate of FDA approvals is constantly decreasing and many resources and time are needed for conventional drug development, the combination of omics data with in silico and experimental drug repurposing approaches can be used for the repositioning of FDA-approved drugs against druggable protein targets in CLL. All this information could be further integrated with other available data (clinical, pharmacovigilance, basic research) to prove the biological/clinical significance, as well as the rational existence of these findings. This review emphasizes both proteomics and drug repurposing approaches. Proteomics provide essential multi-level information on the structure and function of the whole proteome under specific conditions, which is closer to the actual phenotype of the biological system. Different state-of-the art proteomics approaches can unravel the complex and heterogeneous CLL molecular phenotype, providing new insights on the mechanisms of its initiation and progression, the identification of protein biomarkers and putative drug targets for drug repurposing for more effective therapeutic options. On the other hand, drug repurposing approaches are promising faster and more precise novel pharmaceutical strategies in comparison to traditional drug discovery approaches that could enhance the drug arsenal in CLL treatment.

## 2. Application of State-of-the-Art Mass Spectrometry-Based Proteomics in CLL Studies

### 2.1. The Powerful MS-Based Proteomics

Extensive research studies to characterize human molecular physiology in health and diseases have mostly focused on genomics, epigenomics and transcriptomics-based analyses, providing a prediction of a given cellular condition, overlooking proteins, the main effectors of cell phenotype and progression. The proteome is highly dynamic, fluctuating both spatially and temporally, mainly due to various endogenous and exogenous signaling events that regulate gene expression, protein maturation, structure, function and other mechanisms, including alternative splicing or/and post translation modifications (PTMs), that enhance proteome diversity and dynamics, producing, by far, a larger number of proteoforms than the predicted number of genes in a cell. Proteomics enables the large-scale characterization of the complete proteome of a cell, tissue, biological fluid, or organism, employing mainly state-of-the-art mass spectrometry (MS)-based and bioinformatics approaches. Thus, proteomics represents the best approach to assess major aspects of cellular biology in health and disease. Advances in the field allow approaches for the global or targeted comparative proteome and phosphoproteome profiling, the accurate detection of PTMs, and the analysis of protein interactions under a specific, well-defined set of conditions of interest [55]. Technological advances in the field, nowadays, allow the assessment of the whole proteome of complex eukaryotic cells in one experiment in a few hours. Methodological innovations allow multiplexing by enabling the simultaneous analysis of multiple samples in a single run, greatly improving the analytical power of the method. These advancements have made proteomics one of the most rapidly developing fields of cell and molecular biology [56,57,58]. The study of the proteome represents an invaluable piece of information for understanding complex features and mechanisms of the pathogenesis of diseases, including cancer. As the proteome reflects the physical condition of a patient at a specific time point, the proteomic data may enable better decisions on how to treat such a patient. Hence, proteomics represents a fundamental method enabling precision medicine for all patients worldwide [59].

### 2.2. Revelation of CLL through Proteomics

CLL pathogenesis is an outcome of both genetic predisposition and environmental impact, which generates extreme heterogeneity in disease behavior and clinical outcomes [55] that is reflected in the proteome of the patients. Traditionally, proteins involved in the progression of the CLL have mostly been studied using conventional biochemical approaches, focusing on one study of a single protein or a small group of proteins [60] providing significant mechanistic details and correlations, but failing to address the system-wide molecular and biochemical complexity of CLL. Nowadays, proteomics approaches have enabled the high-throughput investigation of the significantly altered abundance of proteins, their modifications, their topology, their function, structure, and interactions in CLL, offering valuable information on the disease regulation and progression, connecting the missing links of the available information. The next paragraphs review all the currently available proteomics studies in CLL and address how this data have been employed to understand the complex molecular mechanisms involved in CLL and identify novel therapeutic targets and biomarkers for diagnosis and prognosis.

### 2.3. Proteomic Studies in CLL

In the last two decades, more than 44 proteomics studies have been performed on CLL to identify various changes on the proteome correlated to the molecular pathogenesis of the disease with the potential to serve as biomarkers and/or therapeutic targets [55,57,60,61] (Figure S2, Table S3). We have organized these studies in ten categories, based on specific subgroups of patients, samples of analysis, treatments, and comparisons (Figure S2, Table S3). Many studies fall into more than one category. Most of the studies involve primary CLL cells from patients that are compared either each other or/and with healthy primary B cells. The challenge is how to combine these studies to identify the most prominent protein signature candidates and exploit such changes in the proteome therapeutically.

#### 2.3.1. Proteomic Studies Associated with IGHV Mutational Status

There are twelve proteomic studies that concern the impact of IGHV mutation on the molecular pathogenesis of CLL in purified primary cells from patients. In the vast majority of the studies, primary cells from patients with M-CLL were compared with primary cells from patients with UM-CLL, and in some cases with healthy primary B cells. The first one from Cochran et al. studied the total proteome from six M- and six UM-CLL patients and reported four proteins (nucleophosmin, F-actin-capping protein beta subunit, 14-3-3 beta protein, laminin-binding protein precursor) that showed reduced abundance in UM-CLL [62]. Barnidge et al. performed a similar comparative analysis on primary cells from two patients on two different sub-cellular fractions (cytosol and membranes) and found 13 proteins exhibited differential abundance in the two samples [63]. They focused on Cytochrome c oxidase polypeptide VIb (COXG) that was found reduced in UM-CLL and may provide evidence of an altered mitochondrial protein expression in this subset of patients. Rees-Unwin et al. detected differentially expressed proteins between four M- and four UM-CLL patients and, in contrast to previous findings, nucleophosmin 1 showed increased abundance in UM-CLL patients [64]. They also proposed that nucleophosmin 1 is associated with ribosome components and undergoes a nucleo-cytoplasmic shuttling, signifying altered protein biosynthesis in UM-CLL. Eagle et al. compared the total proteome between nine M- and nine UM-CLL cases [65] resulting in 274 differentially regulated proteins between the two subsets of samples. The most differentially expressed proteins detected at lower abundance in UM-CLL were associated with cell migration/adhesion pathways (impaired Rap1-dependent αLβ2-mediated migration) and cytoskeletal remodeling (disfunction of S1PR1), while proteins involved in transcription and translation (LEF-1) were up-regulated. Recently, Thurgood et al. investigated proteomic differences between six M- and six UM-CLL vs. healthy B cells, after the fractionation of cytoplasmic and membrane proteins [66]. In total, 349 and 193 proteins were differentially regulated in CLL vs. healthy B cells, as well as in M- vs. UM-CLL, respectively. Differentially regulated biological processes between the two subtypes were cell migration (ITGB1, DESG1, many chemokines), BCR signaling, transcription and translation, and cell proliferation (RAP1A, CAT) in accordance with the previous findings. Biological processes also included stress pathways (e.g., the detoxification of ROS), telomerase regulation, intracellular trafficking (FKBP4), other key signaling pathways (integrin signaling, PI3K, chemokine/cytokine, ATR, VEGF) and metabolic pathways (glycolysis/gluconeogenesis, pyruvate, glutathione, sphingolipid signaling, pentose phosphate pathway) (e.g., GAPDH, GLUT2, NAXE). They focused on the altered metabolic pathways and supported the association of poor clinical outcome with the stimulation of metabolism in UM-CLL.

In a pilot study on a limited number CLL patient samples and normal B cells (3 M vs. 3 UM), Eagle et al. created a CLL-specific spectral library employing state-of-the-art data-independent acquisition (DIA)-MS technologies, such as SWATH (Sequential Windowed Acquisition of all THeoretical fragments) [67]. The inclusion of normal B cells in the library allows the future comparative analysis of malignant vs. normal B cells, and at the same time portrays differences in the B cell proteome from early stages of the disease.

There are also proteomics studies that investigated the differences in the proteome between M- and UM-CLL as a side project. Scielzo et al. found a prominent increase in the phosphorylated form of the hematopoietic lineage cell-specific protein 1 (HS1) in UM-CLL [68]. In accordance with this finding, Perrot et al. verified that the phosphorylated form of HS1 is mostly present in UM-CLL cells [69]. Alsagaby et al. detected acinus required for apoptotic chromatin condensation following activation by caspase 3 reduced in UM- vs. M-CLL [70]. Glibert et al. found that most of the differentially abundant proteins in M- vs. UM-CLL are involved in metabolic processes or they are cytoskeletal, as previous reported [71]. Moreover, Johnston et al. found 149 up-regulated proteins and 127 down-regulated proteins in UM-CLL that could be used as biomarkers differentiating CLL subtypes [56]. On the other hand, Díez et al. showed no significant differences in global protein profiles concerning the mutational status of IGVH genes [72].

#### 2.3.2. Proteomic Studies Associated with BCR Signaling

There are only three proteomics studies that investigated BCR signaling. Perrot et al. performed a time-course comparative proteomic analysis to study the effect of BCR signaling activation through anti-IgM stimulation in three UM- and three M-CLL samples [69]. In the UM-CLL samples, 25 proteins showed significant differential protein abundances after BCR activation, compared to the six altered protein abundances in the M-CLL samples. The 25 proteins were involved in cytoskeleton activity, cell growth, apoptosis, metabolism, and signal transduction. The most interesting among them were programmed cell death protein 4 (PDCD4), UV excision repair protein (RAD23B) and lymphocyte-specific protein-1 (LPS1) that were down-regulated in UM-CLL cells after BCR activation, as well as heterogeneous nuclear ribonucleoprotein K (HNRNPK) that was up-regulated in UM-CLL. Kashuba et al. studied proteomics alterations induced by the stimulation of BCR on three CLL samples [73]. They found that, among the 16 proteins that were differentially expressed, low molecular weight kininogen (LMWK) was up-regulated in all three samples, confirmed by immunoblotting, indicating a role of this protein in BCR signaling of CLL cells. Díez et al. proposed a novel strategy to conduct peptide sequencing of surface immunoglobulin (sIg) and other immune-related proteins of CLL cells [74]. Briefly, they evaluated three different sample processing methods for peptide sequencing of BCR belonging to B-CLL cells (nine CLL patients) and they identified a total of 98, 60 and 426 unique peptides, respectively. Only 15 peptides identified were common in the studied strategies, confirming the importance of the method followed, as well as the importance of the integration of both genomics and proteomics for a full characterization of Ig sequences, BCR and immune system-related proteins.

#### 2.3.3. Proteomics Insights into Cytogenetics and Driver Mutations

There are four proteomics studies focusing on the effect of cytogenetics and driver mutations on the B cell proteome that drives CLL initiation and progression. Voss et al. analyzed the proteomic profile of 24 CLL patients with defined chromosomal characteristics (del11q22-q23, del13q14 or del17p13), and identified 16 differentially enriched proteins that predicted chromosomal aberrations and overall survival [75]. Enzymes that are implicated in ROS detoxification (e.g., thioredoxin peroxidase 2, glutathione S-transferase) were found in decreased abundance. Moreover, protein disulfide isomerase precursor showed reduced levels, while heat shock protein 27 (HSP27) was increased in the patient groups with short survival times (e.g., del17p13). Díez et al. utilized an antibody microarray assay integrated with an MS/MS strategy, to examine the impact of del13q14, del17p13, trisomy 12, and NOTCH1 mutations on the expression of 224 signaling proteins, in a cohort of 14 newly diagnosed B-CLL patients (vs. 63 healthy controls) [72]. Protein kinase C (PKC) family members were identified as down-regulated in nearly 75% of the samples. Moreover, del13q14 was linked to the up-regulation of JUN and cyclin A, B1 and D1, and to the down-regulation of PRKC and RAF1; del17p13 was linked to the up-regulation of CDKN2A, and JNK and to the down-regulation of p21 CDK inhibitor. NOTCH1 was associated with the up-regulation of CDK4 and 6, JUP and MYH9 and the down-regulation of cyclin B1, PKCγ, p21 CDK inhibitor, β-synuclein, CFL1, HDAC1, PPP1CA and PRKCB. Trisomy 12 showed no significant differences at the proteome level. Huang et al. also showed that many patients with 13q- deletion clustered in one group [76]. Bretones et al. explored altered patterns of global protein synthesis and translational fidelity in RPS15-mutated CLL [77]. They performed comparative proteomics analysis of different clones of GFP-RPS15WT, GFP-RPS15P131S, and GFP-RPS15S138F in HEK293T cells with partially or totally ablated endogenous RPS15, and they revealed two strikingly different phenotypes. Additionally, they expanded their studies to five mutations (GFP-RPS15^P131S^, GFP-RPS15^G132S^, GFP-RPS15^T136A^, GFP-RPS15^H137Y^, GFP-RPS15^S138F^) in a CLL-specific cell line (MEC-1) with ablated endogenous *RPS15*. In total, 6640 proteins were quantified, and all the variants were clustered. They suggested that RPS15 variants, which hold a prominent role in CLL pathobiology, may induce the up-regulation of RNA translation and peptide chain elongation, as well as a metabolic shift at the proteome level, in both HEK293T and MEC-1 cells (the down-regulation of pyruvate metabolism, TCA cycle, respiratory electron transport chain and metabolism of lipids and lipoproteins).

#### 2.3.4. Proteomic Approaches for Profiling Histones

Epigenetic events involving the role of histones’ regulation are crucial for CLL development and progression. There are three proteomic studies profiling histones in CLL focused on H2A variants, the most significant differentially expressed histones of the disease. Su et al. investigated the differences in histone isoforms distribution in 40 CLL patients (vs. four controls). The group proposed that the decreased abundance of histone H2A variants (H2AFL, H2AFA/M) that was seen in primary CLL cells vs. normal B cells, could be used as a clinically significant biomarker for CLL diagnosis [78]. Glibert et al. also analyzed the expression of histone variant H2A in 12 CLL samples (six M- vs. six UM-CLL) and healthy controls. The authors proposed that a truncated/proteolytic product of the H2A (cH2A) may be found up-regulated in CLL patient samples [71]. However, after a thorough analysis in various cancer cell lines, cH2A was rejected as a potential biomarker, as it was found to be a product of contaminating myeloid cells that were co-isolated with the lymphoid cells. Extending the characterization of the histone proteome of CLL samples, Singh et al. applied a proteomic approach on isolated histones from 87 CLL patients vs. 5 healthy volunteers, to identify tissue specific patterns of histone PTMs (vs. bladder and breast cancer). They showed that H2A is the most clinically relevant isoform, since patients with a detectable level of H2A had a significantly shorter time to treatment [79].

#### 2.3.5. Deciphering Cancerous Microenvironment Interactions through Proteomics

They are four proteomics studies focusing on the role of the cancerous microenvironment in CLL development and progression. O’Hayre et al. performed the first proteomic study investigating the microenvironment’s interactions. They focused on the effects of one chemokine, CXCL12, known to be secreted by NLCs and interacting with its receptor, CXCR4. The latter is found to be overexpressed in CLL cells, supporting survival and drug resistance [80]. Phosphoproteomics analysis of unstimulated and CXCL12-stimulated primary CLL cells identified two novel downstream targets of CXCL12/CXCR4 signaling. PDCD4, a tumor suppressor, and HSP27, an anti-apoptotic factor, were both found to be up-regulated in CXCL12-stimulated cells and could become possible druggable targets. Prieto et al. investigated the plasma-derived exosomes secreted in CLL, which in turn also participate in the continuous crosstalk between the tumoral cells and their microenvironment [81]. Briefly, they studied the proteomic profile of exosomes from five patients with progressive disease and five patients with indolent disease, both at the onset of disease and during its progression. They highlighted networks specific for leukemia progression related to cell infiltration, tumor proliferation, the PI3K/AKT pathway, survival and apoptosis, inflammation, and oxidative stress, and they focused on the proteins S100-A9 and junction plakoglobin (JUP), activators of the NF-κB and Wnt pathway, respectively. They further analyzed and proved the predominant role of S100-A9 in exosome secretion and CLL progression. Haderk et al. also investigated the proteome profiling of plasma-derived exosomes in CLL [82], between four CLL patients vs. four healthy donors. As a result, 91 proteins were significantly differentiated in the exosomes of CLL vs. healthy donors, including various vesicle markers (annexins, actin- and Ras-related proteins, 14-3-3 signaling proteins) elevated in CLL-derived exosomes, implying an altered composition of plasma exosomes in CLL. Mangolini et al. studied plasma membrane profiling of primary stromal cells derived from mouse embryonic livers, in the presence and absence of Notch2, showing that malignant B cells are responsible for the activation of Notch2 signaling in bone marrow stromal cells (BMSCs) [83]. They identified 1055 plasma membrane proteins that showed different clustering patterns in stromal cells’ monoculture in comparison to coculture with CLL cells, as well as in the presence or absence of Notch2. Notch2 was identified as the most differentially regulated cell surface protein, explaining the 36 differentially expressed Notch2-regulated proteins in stromal cells. The main conclusion from this study is that Notch2 activation in the microenvironment is required for the activation of canonical Wnt signaling in tumor cells, through the inhibition of GSK3-β and the stabilization of β-catenin.

#### 2.3.6. Subcellular Proteomics Studies

A widely used approach to reduce sample complexity and gain detailed information on protein localization and translocation is sub-cellular fractionation into different compartments and organelles. The produced biological sample contains highly enriched, pure subcellular organelles, with well-preserved structure and functions, facilitating the detection and further analysis by proteomic approaches of otherwise difficult-to-study proteins (e.g., transmembrane, chromatin-related, low abundant). There are currently five subcellular proteomics studies in CLL shedding light on proteome cellular localization.

Membrane proteome: Boyd et al. studied the CLL plasma membrane proteome for the identification of new potential therapeutic targets [84]. They identified 500 proteins, with proteins recognized for first time to be resident in the CLL plasma membrane. They focused on two novel proteins (MIG2B, BCNP1) with probable plasma membrane localization. BCNP1 was found to have three predicted transmembrane domains, and MIG2B was found to contain the plasma membrane-binding ERM domains. They also examined their mRNA abundance in multiple normal tissues, as well as in B cell malignancies. BCNP1, in contrast to MIG2B, was found to be a B-cell-specific protein and its role in the pathogenesis of CLL remains to be determined. Miguet et al. characterized the plasma membrane proteome derived from three types of hematological malignant cells (CLL, small cell lymphoma—SLL and mantel cell lymphoma—MCL) questing proteins with potential discriminatory value between MCL and the two other diseases [85].

Barnidge et al. performed proteomics analysis of two patients’ samples (UM- vs. M-CLL) on two different sub-cellular fractions (cytosol and membranes) and found a reduced abundance of the COXG in the membrane extraction (UM-CLL) [63]. Mangolini et al. studied plasma membrane profiling of EL08-1D2 cells monoculture/coculture with CLL cells, ±Notch2 (Notch2WT/Δ. Notch2) and found the pathways were regulated by Notch2 [83]. Thurgood et al. analyzed the cytoplasmic and membrane proteomic profile in six M- vs. six UM-CLL vs. three healthy donors’ samples [66]. They found an increase in CXCR3, ABCF3, and a decrease in IGKC in the membrane fraction of CLL patients compared to healthy donors.

Nuclear proteome: Henrich et al. studied the differentially expressed nuclear proteins in hemopoietic cell lines to find nuclear proteomic profiles aiding the classification of leukemia subtypes [86]. In MEC1 (CLL), nuclear prohibitin and HSP70C demonstrated increased levels, while nuclear ribonucleoproteins snRNP F, hnRNP H3 and hnRNP K, proliferating cell nuclear antigen (PCNA) and heterogeneous nuclear ribonucleoprotein D0 (AUF1) demonstrated decreased levels, compared to HL-60 (AML), CCRF-CEM (T-ALL), and Raji (BL). Mayer et al. also studied the nuclear fraction of CLL patients in comparison to healthy donors and found 441 proteins to be significantly altered. Among them TP73, KIAA0907, PTPN2, DNPH1, LEF1, HIVEP1, HMG20B and ALOX5 were found to have increased abundance, while NPRL2, JARID2, DAPK3 and HIVEP3 were found with reduced abundance. They combined these results with the cytoplasmic fraction to draw conclusions [87].

Cytoplasmic proteome: Gez et al. compared the cytoplasmic proteome profiles of four cell lines of lymphoma and leukemia, including MEC1, and found 29 cytosolic proteins differentially expressed in the MEC1 cell line [88]. Among them, C-1- tetrahydrofolate synthase, MTHFD1, elongation factor 2, β-tubulin, transgelin-2 and stathmin were up-regulated in MEC1 and Raji (BL), compared to HL-60 (AML) and CCRF-CEM (T-ALL). They proposed that these proteins may play a role in B cell development. Barnidge et al., performing a proteomics analysis on the cytosol and membranes of two patients’ samples (UM- vs. M-CLL), found an increased abundance of A32A in the cytoplasm (UM-CLL), without, however, confirmation of the result in a following cohort study [63]. Mayer et al. also studied the cytoplasmic fraction of CLL patients vs. healthy donors and found 426 proteins to be significantly altered. Among them, TAX1BP1, ZNF207, CKAP4, STAMBPL1, HMOX1, ATOX1, ID3, CCDC88A and BCL2 were found with increased abundance, while HHEX, PNN and LAIR1 were found with reduced abundance. They combined these results with nuclear fraction to draw conclusions [87]. Thurgood et al. performed proteomics analysis in the cytoplasmic and membrane fractions of six M- vs. six UM-CLL vs. three healthy donors’ samples [66]. In the cytoplasmic fraction, they found an increase in HMGN2, PKM, GAPDH, CALR, HMGN, H3F3A, HMGN4, CLTA, ACLY, NUTF2, IDH2, TXNRD2, HIST1H4A, AIFM1, MDH2, CDC5L, as well as a decrease in FAM107B, HNRNPUL1, PLIN3, CBR1, PNP, AK1, MIF, CAPZA2, CES1, SOD2, CDC42, SPRYD4, RPS21, HPRT1 and WARS in CLL patients compared to healthy donors.

#### 2.3.7. CLL Prognostic and Diagnostic Biomarkers by Proteomics Analysis

Almost half of the above-mentioned studies (22) (Table S3) investigate, among other studies, potential diagnostic and prognostic biomarkers for the disease. Scielzo et al. studied the total proteome of 14 CLL patients with either poor or good prognoses and found a differential expression of HS1 between the two patient subsets [68]. Specifically, patients with poor prognoses mostly had a constitutively phosphorylated HS1 protein, in contrast to patients with good prognoses that was validated in a larger cohort of patients (26), suggesting a constant stimulation possibly triggered by a recent/persistent BCR-mediated activation. They proposed HS1 as a prognostic CLL biomarker and a possible new molecular target for the treatment of patients with aggressive disease. Furthermore, Miguet et al. analyzed 39 CLL and 20 control serum samples and noted a novel increase in the sulfite form of transthyretin in the patient group [89]. Alsagaby et al. analyzed 12 samples deriving from segregated patients based on CD38, ZAP70, as well as IGHV mutational status, to detect proteomic differences of prognostic value in B cell CLL. The group identified 728 proteins, among which four proteins (increased expression of TRAP150, TCL-1, S100A8 and reduced expression of MYH9) were associated with high-risk CLL [70]. Huang et al. identified 84 differentially expressed proteins as putative biomarkers for progressive CLL by analyzing the cell proteome of 77 CLL patients [76]. Specifically, they found decreased levels of prohibitin, PARP1, RhoA, Rac2, MYH9 and DHE3, as well as an increased abundance of TIF1B, ANP32A, HIST1H1C, SET, and of heat shock proteins, CH10 and CH60, in progressive CLL. Johnston et al. attempted to characterize expression across the whole proteome of CLL [56] in 14 CLL vs. 3 healthy donor samples. In total, 544 proteins were up-regulated, and 592 proteins were down-regulated in the malignant cells. Down-regulated proteins included cell adhesion molecules, such as integrins, and suggested a reduced capacity for endothelial transmigration. Among the up-regulated proteins in CLL, there are some already established hallmarks of CLL, e.g., CD5, BCL-2, ROR1, and CD23. Furthermore, there are several previously unrecognized surface markers, e.g., cytoskeleton-associated protein 4 (CKAP4) [87], polymeric immunoglobulin receptor (PIGR) [87], transmembrane and coiled-coil domain protein 3 (TMCC3), CD75, and surface proteins (e.g., LAX1, CLEC17A, ATP2B4), involved in BCR signaling. Moreover, 95 of the up-regulated proteins in CLL cells were related to mRNA processing and splicing (splicing factors, pre-mRNA splicing factors, small nuclear ribonucleoproteins and heterogeneous nuclear ribonucleoproteins). Furthermore, the most significant up-regulated potential targets, e.g., G2 checkpoint kinase (Wee1), heme oxygenase isoforms (HMOX1/2), histone deacetylase (HDAC7) and inositol 4-phosphatase (INPP5F), were annotated to known small molecular inhibitors as a potential treatment strategy.

In Table S3b–d, from all these studies, we have collected more than 219 proposed biomarkers or/and drug targets including 89 prognostic and 141 diagnostic.

#### 2.3.8. Specific Proteome Signature of CLL vs. Normal B Cells or Other Diseases

There are ten proteomic studies that compared the CLL to normal B cells’ proteome. Mayer et al. employed state-of-the-art proteomics for the investigation of aging-associated B cell proteome changes (116). Peripheral B cell cytoplasm/nucleic fractions from six healthy donors (three elderly, three younger), and nine B-CLL patients were analyzed via shotgun proteomics. The results were compared to proteomics data of cytoplasm/nucleic fractions of chronic B cell leukemia cell line JVM-13, finding a rather poor relation to both primary B cells and B-CLL cells. Principal component analysis on the 6945 identified proteins separated these four groups, showing B cells of age-matched controls laying between those of young donors and CLL patients. B cells from aged controls displayed significant alterations of proteins related to stress management in mitochondria and during ROS stress (DLAT, FIS1, NDUFAB1), and DNA repair (RAD9A, MGMT, XPA) in comparison to young donors. These alterations are correlated with the aging of B cells and may also have a role in tumorigenesis. Moreover, B-CLL cells demonstrated unique features, including the loss of tumor suppressor molecules PNN and JARID2, the stress-related serotonin transporter SLC6A4, and the high expression of proteins associated with stem cell phenotype (ZNF207, CCDC88A, PIGR, ID3).

There are more than nine proteomics studies that performed comparative analysis of CLL vs. normal cells and almost seven proteomic studies that compared CLL proteome with other diseases [56,66,71,78,79,82,89,90,91]. Marina et al. used a ProtoArray for analyzing CLL vs. CML samples. They focused on the methodology and the sensitivity of the method, without focusing on the biological results [92]. Schröder et al. also screened antibody microarrays for identifying marker proteins in the plasma of 61 DLBCL, 19 CLL, and 20 follicular lymphoma (FL) samples compared to 100 age- and gender-matched controls [90]. They showed that CLL exhibited the most differentiating pattern of the three diseases, while the most differentially expressed proteins in CLL were involved in the regulation of programed cell death. Moreover, they proposed some possible biomarkers for CLL, such as TSN16, NPT1, PCNA, CATD, TNFA, TNR6 (FAS), TR10C, IL8, and VCAM. Johnston et al. detected proteomic differences in BL and CLL in mouse models using quantitative proteomics [91]. Eμ-TCL1 tumors (CLL) showed up-regulated ER stress response proteins and signaling components, including both subunits of the interleukin-5 (IL5) receptor, whereas Eμ-TCL1 plasma contained increased proteins of immune-response, inflammation and microenvironment interactions, with putative biomarker candidates for early-stage cancer (e.g., haptoglobin, nucleolin, 60S ribosomal protein L35, heat shock 70 kDa protein 4, 60S ribosomal protein L23a, inter alpha trypsin inhibitor heavy chain H1). Recently, Khodadoust et al. investigated tumor-derived class I (MHC-I) and II (MHC-II) antigen-presentation profiling of six FL, one DLBCL, and two CLL samples, using immunoprecipitation followed by MS [93]. They found the presentation of the clonal immunoglobulin molecule, including neoantigens by both class I and class II MHC, though more commonly in class II MHC. These findings suggested that immunoglobulin neoantigens are presented across most subtypes of B cell lymphomas and these neoantigens could become possible targets in immunotherapies. Henrich et al. studied the nuclear proteome of CLL, T-ALL, AML and BL [86], and Gez et al. the cytosolic proteome of CLL, T-ALL, AML and BL [88], while Miguet et al. [85] studied the plasma membrane proteome of CLL, SLL and MCL.

#### 2.3.9. Proteomic Analysis after Pharmaceutical Treatment

There are six studies that analyzed CLL’s proteome before and after treatments. Henrich et al. studied the action of fludarabine nucleoside (2-FaraA) in specific nuclear proteins [94]. As a result, 15 nuclear proteins changed in abundance by > two-fold, e.g., calmodulin, prohibitin, β-actin variant, and structure-specific recognition protein were up-regulated. Down-regulated proteins were 60S ribosomal protein P2B, fumarate hydratase, splicing factor arginine/serine-rich 3, and replication protein A2. Che et al. studied the proteomic effects of the Hsp90 inhibitor, SNX-7081, on the p53-mutated B cell CLL cell line, MEC1 (nuclear, cytosolic and mitochondrial fractions) [95]. They detected decreased levels of DDB1, MCM2, c-Myc, PCNA and increased levels of pRb and cyclin D1, that were also confirmed in other types of hematopoietic malignancies. Kaufman et al. also studied MEC1 cells treated with SNX-7081, alone and in combination with 2-FaraA, using quantitative shotgun proteomics [96]. They confirmed the reduction in MCM2, MYC and PCNA, as well as the activation of cyclin D1 caused by SNX-7081. They showed an increased abundance of proteins involved in nucleobase, nucleoside, nucleotide and nucleic acid metabolism, including the DNA damage protein TOP2A, and proteins positively regulating DNA replication and repair, after 2-FaraA treatment. They concluded a synergy between SNX-7081 and 2-FaraA, as 2-FaraA induces DNA damage and SNX-7081 down-regulates DNA repair proteins, resulting in the accumulation of DNA damages in the cells, which initiates apoptosis. The synergistic effect may be mediated by a SNX-7081-induced loss of MYC and NFkB2 p100 accumulation. Moreover, they focused on the induction of the DNA damage marker, γH2AX, which was found to also be increased in other p53-mutated human B-lymphoid cancers after treatment with SNX-7081 and 2-FaraA. Furthermore, Kruse et al. selected a set of 13 compounds consisting of pan-kinase tool inhibitors and clinical multi-kinase-targeted drugs and investigated their impact on the viability of CLL cells [97]. They found that two CDK inhibitors, BMS-387032 and flavopiridol, caused a dramatic reduction in viable cells, and that BMS-387032 does not exhibit toxicity to healthy cells as much as flavopiridol does. They discovered the proteomic targets of these inhibitors by a kinobeads competition assay (in comparison with staurosporine that was used as a positive control) and found that these drugs deregulate the positive transcriptional elongation factor (p-TEFb) complex, indicating a critical role of p-TEFb inhibitors in CLL clinical treatment. Beckmann et al. examined the (phospho)proteome of primary CLL cells after ibrutinib treatment, finding higher basal phosphorylation in UM-CLL [98]. One of the differentially expressed proteins between the two subtypes, MARCKS, which was highly phosphorylated after BCR stimulation and reduced by BTK inhibition, was proposed to be a possible biomarker for this kind of treatment.

#### 2.3.10. Drug Repurposing Based on Proteomic Studies in CLL

Only one from the above-mentioned proteomic studies in CLL resulted in druggable targets and repurposed drugs [56]. They used pathway analysis to annotate potential therapeutic targets based on existing drug/inhibitor knowledge. Subsequently, they made a list of the 20 most consistently up-regulated annotated targets of small molecular inhibitors in CLL. Nevertheless, they have not further investigated those inhibitors. There is also another study mentioned above, following a reverse course, which profiled a panel of clinical multi-kinase inhibitors for their ability to induce apoptosis in primary CLL cells. They used the kinobeads proteomics method to investigate the targets that were most potently affected [97].

#### 2.3.11. Reverse-Phase Protein Array (RPPA) Studies in CLL

RPPA analysis has also been used to identify proteins and pathways involved in CLL pathobiology. Shull et al. carried out a RPPA study in 18 CLL patients and 6 healthy CD19+ B cell protein extracts, where they detected up-regulated AKT/mTOR-related proteins in CLL, i.e., eIF4G and phosphorylation at serine 65 of 4E-BP1 [99]. Based on these results and on the greater apoptotic effect from inhibitor treatment comparisons using the dual PI3K/mTOR inhibitor NVP-BEZ235 vs. mono BTK inhibitor (Ibrutinib) or PI3Kδ inhibitor (Idelalisib), the group emphasized the possible central role of mRNA translation in CLL survival, depicting its potential as a CLL therapeutic target. Frezzato et al. performed an RPPA investigation in B lymphocytes from 57 CLL patients and 11 healthy individuals, identifying several proteins significantly altered in CLL vs. healthy B cells, highlighting, among others, a decrease in apoptosis-related proteins (cl-caspase-7, cl-caspase-9) and increased levels of HSP70 and Smac/DIABLO [100]. The group testing the efficacy of HSP inhibition combined with other drugs (i.e., fludarabine, 17-DMAG) reported an enhanced apoptosis induction in CLL, supporting the use of the inhibition of HSP70 alone or in combination to treat CLL. Patel et al. performed a proteomic profiling of pre- and post-acalabrutinib-treated CLL patients using RPPA technology [101]. They reported that acalabrutinib treatment changed protein levels in signaling, cell cycle, transcription, translation, and metabolism-related proteins, decreased total BTK, and increased Bcl-2 family proteins. Using venetoclax combined with acalabrutinib had a synergistic effect; increased Bcl-2 (venetoclax’s target protein) suggested a role for the increase in venetoclax-induced cell death after acalabrutinib treatment, encouraging the combinatorial possibilities of acalabrutinib therapy. Finally, the study of Vangapandu et al. analyzed CLL cells before and after coculture with stromal cell lines using RPPA, identifying several altered protein levels involved in signaling pathways, cell cycle, transcription, and translation regulation (including STAT3, NF-κB, PDGFRβ, PAI1, cyclin B1) [102]. Furthermore, the group proposed caveolin-1 as an interesting, significantly up-regulated protein in CLL, of putative therapeutic interest.

## 3. Drug Repurposing in CLL

### 3.1. Drug Repurposing in Hematological Malignancies—The Performance of CLL

Drug repurposing is a strategy of identifying new therapeutic uses for pre-existing, FDA-approved or investigational drugs, that are outside the aim of the original medical indication. This approach is based either on the fact that different diseases may have similar molecular signatures and druggable targets, or that off-target drug effects may be useful for the treatment of other diseases (polypharmacology) [103,104,105]. In comparison to conventional drug discovery, the main advantages of this process are that it requires almost half the years and one third of the money needed in the first method [106], while the safety and toxicity profile of repurposed drugs is completely established.

Even increasing data from multiple experimental studies and clinical observations have depicted that different non-neoplastic drugs have potential anticancer activity, including cardiovascular drugs, antipsychotics, antidepressants, microbiological agents, anti-viral drugs, antibiotics, non-steroidal anti-inflammatory drugs, antidiabetic, anti-emetic drugs, etc. [107,108,109,110,111,112]. There are many repurposed drugs that are already used in hematological malignancies. The discovery of these drugs is based on (i) clinical trials results, (ii) random observations, (iii) the biological background of a disease, or (iv) in vitro and (v) in silico high-throughput screenings [107]. There are also many studies investigating drug repurposing in different subtypes of hematological malignancies [113,114,115,116,117,118,119,120,121]. Concerning CLL, 2816 compounds were studied in vitro and 102 of them influenced the lymphocytes of all six CLL patients tested. Only five of them (auranofin, azacytidine, dimercaprol, podofilox, plicamycin) had no simultaneous effect on the respective cells of the five healthy volunteers, used as the control group [122]. Additionally, there are studies that support the repositioning of nelfinavir and chloroquine as a combinatorial therapy, as well as FDA-approved allergy medications (e.g., clemastine) with ibrutinib and roflumilast with idelalisib. These drug combinations showed great anti-cancer properties in CLL [123,124,125]. In Table 1, all the repurposed drugs in CLL derived either from proteomics studies or/and other studies are summarized.

### 3.2. Drug Repurposing Methodologies

Drug repurposing approaches that are currently used vary and are mainly divided into two main categories: computational (in silico) and experimental (in vitro) methods [127].

#### 3.2.1. In Silico—Computational Analysis

In silico drug repurposing analysis is typically based on (i) the molecular signature of a disease of interest, on (ii) a specific molecular target of interest common in many different diseases, on (iii) a specific drug of interest that can simultaneously affect many different pathways, or on (iv) bibliographic databases concerning drug similarities and general assumptions regarding possible repositionable drugs [128,129]. The data, for the implementation of these methods, are provided by large international databases available that collect all the above information, such as (a) those related to genome or other -omics data of the disease of interest, (b) targeted -omics data of specific subcellular particles, (c) -omics data linked with specific pathways, as well as (d) -omics data related to the action of a drug, (e) toxicity data of a drug, (f) clinical trial data and (g) data of the adverse reactions of a drug [129].

#### 3.2.2. In Vitro—Experimental Analysis

In vitro analysis mainly concerns drugs that are already FDA-approved or are licensed for clinical trials and are being massively tested through high-throughput drug screenings. The most common methods for this kind of analysis are phenotype screening and binding assays. In the first one, a variety of drugs are tested in specific in vitro or in vivo disease models, and the most of effective of them are further investigated for their mechanism of action. In the second one, techniques such as affinity chromatography and mass spectrometry are used to identify novel targets of known drugs and/or drugs with a binding affinity in specific targets [127].

#### 3.2.3. Pros and Cons of Drug Repurposing Methodologies

There are many pros and cons in each methodology used. The advantages of in silico approaches are the ability to simultaneously examine large lists of drugs, without many resources and much time needed. Furthermore, through in silico approaches, there is the possibility to understand the pathobiology and development of lymphoid tumors in detail, using data that is already available. The disadvantages of in silico approaches are the reduced reproducibility of the results, in some cases due to the different algorithms and the constantly updating versions, the production of false positive and negative results and consequently, the unsuccessful translation of the results into clinical practice [130,131,132,133,134]. Conversely, the advantage of the in vitro drug repurposing is the tangible proof of the effective action of drugs in an experimental disease model, which consists of the first step of translation into clinical practice. The difficulties of this method are the access to many different drugs, resources, time and effort, as well as the suitability of disease model (e.g., the heterogeneity of cell populations, microenvironmental interactions, species-specific differences) [106].

#### 3.2.4. The Proposed Pipeline

**Figure 2:**
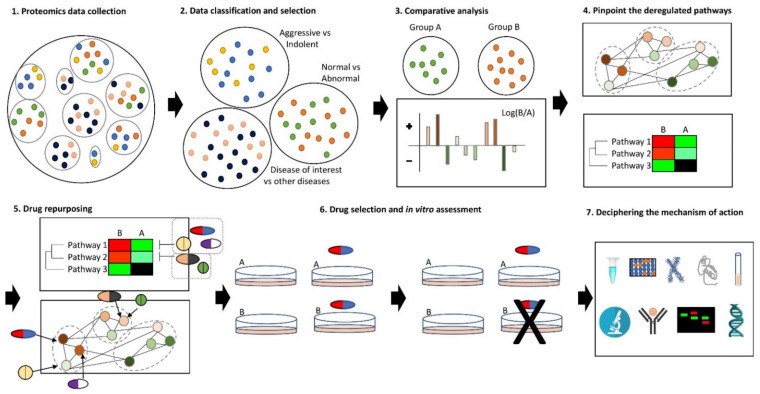
Drug repurposing pipeline based on proteomics data. **1**. Collection of all the available proteomics data of the disease of interest from multiple studies. (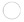: one study and 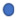, 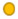, 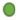, 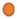, 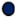, or 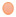: the proteomics data of an individual person, 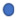: patients with aggressive disease, 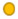: patients with indolent disease, 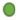: healthy donors, 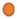: patients, 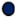: patients with other diseases and 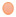: patients of the disease of interest). **2**. Data classification into subgroups with different biological questions that could be compared in the proteome level, e.g., comparison of proteomics data between patients with different stages of the disease, between healthy and patients or between patients with the disease of interest and patients from other diseases; selection of the appropriate group. **3**. Comparative analysis of the proteomics data from the selected subgroup, e.g., healthy donors vs. patients. (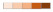 and 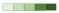: color gradiation of the differences (small → big)). **4**. Translation of the different proteome levels into deregulated pathways, in which these proteins are involved (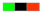: color gradation of the differences (negative → positive)). **5**. Linking of deregulated pathways to FDA-approved drugs documented through their mechanism of action. **6**. Drug selection and in vitro assessment of the drugs both in the appropriate subgroup and in a subgroup control, e.g., cell cultures from healthy donors (A) and patients (B); evaluation of the pharmaceutical effect. **7**. Determination of their molecular mechanisms of action, both expected and unexpected.

Therefore, to safely optimize and accelerate the process of drug repurposing, a combination of these methods is proposed. Effective candidate pharmaceutical agents are first selected from bioinformatic data analyses and then validated for their proposed action and efficacy on in vitro meticulously selected disease models. The ultimate goal of this double-stranding approach is to unravel the pathobiology and development of the disease through the in silico approach, as well as to save resources and time by reducing the list of drugs needed to be tested experimentally and at the same time accelerate the translation of results into clinical practice through the experimental validation and screening of these drugs [131,134]. Consequently, the approach that is usually followed and considered as rationally acceptable is the following:

(1) (Omic) data collection of the disease of interest (proteomics data are preferred).
(2) Data classification and selection depending on the biological question.
(3) Comparison of the data (e.g., with control/healthy donors or other diseases).
(4) Functional and network analysis of the identified differences to unravel the deregulated pathways.
(5) Identification of druggable targets and their association with FDA-approved drugs or drugs licensed for clinical trials (drug repurposing).
(6) Experimental validation that the drugs have the expected properties in a simple disease model (cell culture).
(7) Determination of their mechanism of action and further experimental validation of the drugs on more specific disease models (e.g., 3D culture, in vivo models) [130] (Figure 2).

## 4. Proteomics-Based Drug Repurposing towards Precision Medicine in CLL

The new era of proteomic studies in CLL proposes the usage of the publicly available differential proteome, to find druggable protein targets and propose repurposed drugs against them. All these proteomic data provide insights into CLL pathogenesis, CLL-specific signatures and heterogeneity, and investigate precise diagnostic biomarkers compared to healthy donors and/or other diseases, as well as prognostic biomarkers between different subtypes and stages of CLL patients that could also be used as drug targets for drug repurposing. The most rational and generally accepted pipeline is to (a) select substantial, reproducible and representative data of different subtypes of CLL patients and healthy controls or other diseases, (b) compare these data to detect differentially expressed proteins associated with CLL pathogenesis, (c) evaluate their functional relationship by grouping them into biological processes, pathways and protein interactions networks, and (d) correspond the most important of these differentially expressed proteins to FDA-approved drugs that could potentially rectify the differential expression. Subsequently, (e) the proposed drugs could be validated experimentally for their expected properties, and effectiveness in specific CLL models.

Drug repurposing based on proteomics data can be integrated with precision therapy, providing even more effective therapeutic options to CLL patients [135]. Proteomics data from different CLL patients, who belong to different CLL stages or subgroups, can be compared with each other to identify characteristic differences at the protein level [136,137]. These differences can be biologically and functionally analyzed to find deregulated signaling pathways and biological processes [138,139,140]. Furthermore, co-expression networks can be found between these differentially expressed proteins, and such networks may indicate a common transcriptional regulatory program, a functional relationship or a same pathway or protein complex [141,142]. All these differentially expressed proteins, as well as deregulated pathways and processes found to be biologically important and consist of druggable targets, can be linked to repurposed drugs that already exist to make and expedite individual therapeutic protocols [143].

The repurposed drugs can be further in vitro validated [144]. The first step is to show that the drugs indeed have the expected properties on the patients’ tumor cells that belong to the subgroups that have been investigated, or, if possible, on commercially available cell lines that have the same identities with the patients’ subgroups. The repurposed drugs can be further validated to determine both their targeted mechanism of action and their general molecular consequences in the cells. Subsequently, the minimum effective dose of the drug can be also determined experientially and correlate with the doses already given in clinical practice. Furthermore, drug combinations and synergies that were found to be effective in silico can be also confirmed in vitro in the same way. Drugs and drug combinations found to be effective by the above-mentioned process can be administered to individuals in anticipation of a better effectiveness and reduced toxicity [135,145]. This process can also be repeated during the progression of the disease, based on the new patients’ data. However, more user-friendly, quick, efficient, and affordable proteomic analysis methods, as well as drug repurposing pipelines, are required to apply this method in clinical practice.

**Figure 3:**
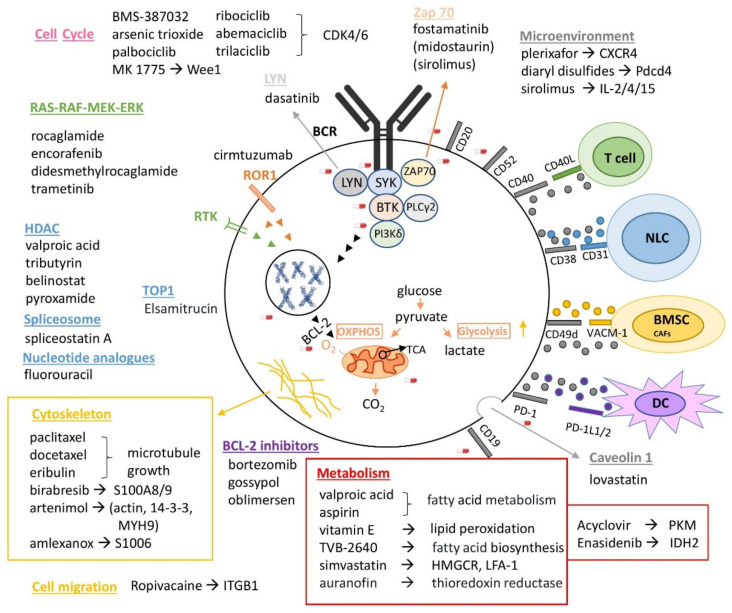
Proposed repurposed drugs in CLL based on both proteomics and drug repurposing data. This figure summarizes the best repurposed drugs candidates in CLL and their mechanism of action, based on both proteomics data and drug repurposing studies. Cytoskeleton remodeling and metabolic pathways (mitochondrial biogenesis/bioenergetics, glycolysis/glyconeogenesis, pentose phosphate pathways, ROS detoxification, pyruvate metabolism, TCA cycle, respiratory electron transport metabolism, nucleotide biosynthetic process) highlighted through the proteomics studies should be considered as two novel categories of therapeutic approaches in the fight against CLL.

In Table 2 and Figure 3, all the proposed repurposed drugs based on proteomics and drug repurposing-derived drug targets using PanDrugs are shown [146].

## 5. Conclusions

Despite the constant research in CLL pathophysiology, ever-increasing diagnostic criteria, and therapeutic pipelines, CLL diagnosis, prognosis and therapy are vague. This creates the need for an even better comprehension of heterogeneous CLL pathophysiology. Omics data have made a significant contribution to this direction, with proteomics data being the main representative, as proteins are the effectors of cellular structure and function, able to gain insightful multilayer information on protein abundance, modifications, and interactions. There are already several CLL proteomic data available, providing great insights to CLL pathophysiology, diagnosis, prognosis and therapy, but these are definitely not sufficient to reveal the complex CLL pathobiology in different conditions and patients. Some of the current pros and cons of the available proteomics studies are summarized below.

Most of the studies involve the proteomic profiling of patient-derived primary cells that are closer to the in vivo patients’ phenotype but are limited by the available number of cells for the analysis. This limitation affects the reliable characterization of low abundant proteins and the detailed study of molecular mechanisms that require a larger number of cells. Nevertheless, primary cells may be less heterogenous if they are properly selected and purified, allowing single cell proteomics. Moreover, primary cells can also be used for ex vivo drug screening and the study of drug mode of action through proteomics.

They are many available comparative analysis studies in CLL showing differences on protein abundancies, but they are only a few comparative proteome-wide analyses on protein modifications, such as phosphorylation, methylation, and ubiquitinoylation, that are not affecting the total protein abundance, but significantly affect the function of proteins.

The majority of the available proteomics studies were performed on a limited number of patients’ samples and cases that need to be further confirmed in a much larger, properly selected cohort of patients. Nevertheless, global proteomics approaches for basic and translational research are mostly performed in a small number of cases, which are carefully selected to represent distinct cases/phenotypes of interest. From these studies, protein candidates that show significant differences in specific types and stages should be used for further validations in larger cohorts using targeted MS- and multiplex ELISA- based proteomics.

The currently available proteomic data in CLL have confirmed and expanded our knowledge on specific proteins and pathways considered as CLL drivers or/and biomarker candidates of initiation, progression, and resistance (Table S3b–e). In addition to a plethora of deregulated signaling pathways, studies on CLL proteomics highlight two main deregulated cellular processes: cytoskeleton remodeling and metabolic pathways (mitochondrial biogenesis/bioenergetics, glycolysis/glyconeogenesis, pentose phosphate pathways, ROS detoxification, pyruvate metabolism, TCA cycle, respiratory electron transport metabolism, and the nucleotide biosynthetic process).

The cytoskeleton remodeling was found to be down-regulated in CLL and especially in U-CLL, in almost all the above-mentioned proteomics studies. Cytoskeleton remodeling involves a signaling cascade that comprises the actin microfilaments, microtubules and intermediate filaments, in order to form protrusions and adhesions required for movement in cell migration [147]. CLL cells have reduced migratory properties to adhere in lymph nodes interacting with the cancerous microenvironment and these properties decrease, inversely proportional with the disease survival (UM-CLL). It is also worth mentioning that proteins involved in cytoskeleton remodeling were found to be secreted in CLL exosomes in accordance with disease progression. Therefore, the inversion of the signature of the proteins involved in this pathway (e.g., S1PR1, actin, 14-3-3s, S100-A9/8, myosin, S100A6), but also of the proteins involved in cell migration (e.g., αLβ2, ITGB1, DESG1) should be considered in novel therapeutic options of CLL.

Several metabolic pathways were found to be up-regulated in CLL and even more up-regulated in UM-CLL. Metabolic pathways’ rewiring is one of the hallmarks of cancer and is already known to have a crucial role in the CLL pathogenesis. The hyperactivation of metabolic pathways in UM-CLL indicate the cruciality of this subtype of disease and is in accordance with the previous pathway, as UM-CLL cells adhere in lymph nodes, interact with the cancerous microenvironment and the microenvironment induces the metabolic activation of them. Proteomics have the advantage of the simultaneous identification and relative quantitation of hundreds of proteins involved in metabolic pathways. In CLL proteomics, a plethora of proteins involved in metabolism were found increased (e.g., GAPDH, GLUT2, NAXE, COX 6B, CPT2, GRPEL1, PKM, FABP3, IDH2, ACADM, ACAA2, CPT2), which could be considered as novel therapeutic targets alone or in combination with other targets.

Other pathways that were found to be differently regulated in CLL, and especially in M- vs. UM-CLL, are transcription, translation, cell proliferation, stress pathways, cell cycle, inflammatory response and other key signaling pathways. Remarkably, one of the known differences in M- vs. UM-CLL cells, which is their response to BCR signaling, was also confirmed through proteomic studies. Among the interesting, deregulated proteins were HMOX1, HDACs, HSP27 and other heat shock proteins that should also be considered as possible therapeutic options.

The above-mentioned proteins and pathways are only a subset of the interesting proteins found in CLL proteomics studies (Table S3b–d). Further exploitation of these proteins and pathways using targeted and untargeted proteomics-based drug repurposing approaches, as described in Section 3.2 and Figure 2, will significantly contribute to the design of precise and efficient treatments in the framework of precision medicine in CLL.

Consequently, even more accurate and reliable proteomic data from many different cases and subtypes of CLL are required, deriving from more cost-effective and user-friendly proteomic techniques to unravel heterogeneous CLL pathophysiology. In addition, integration, meta- and re-analysis of all these proteomics data in a systems biology manner is required to transform them into meaningful and useful knowledge. Therefore, the availability of all proteomics data in public repositories is essential.

The integration of the available proteomic data, their meta-analysis or even re-analysis with improved bioinformatic tools will contribute to a more precise diagnosis, categorization and staging that will allow more effective and less toxic therapeutic pipelines based on drug repurposing. Even more user-friendly, immediate, and faster drug repurposing pipelines would facilitate the alignment of druggable targets identified by proteomics data with FDA-approved drugs. The integration of drug repurposing in precision medicine will be the next therapeutic strategy of CLL, ensuring a long-term survival of CLL patients. To summarize, drug repurposing can be used to:

- Rearrange the already FDA-approved for CLL drugs to be given only in specific molecular traits, avoiding drug resistance.
- Align the drugs licensed for clinical trials in CLL to specific subtypes and stages.
- Find new drugs worthy of further study for the treatment of CLL.
- Make more effective drug combinations that can either target the most deregulated pathways or may have a synergism on a specific pathway/active subnetwork/co-expression network or even show a balancing action on toxicity effects/drug resistance, based both on the molecular signature of the disease and on the networks induced by the administration of the drugs.

Drug repurposing undoubtedly offers multiple benefits in the treatment of CLL patients. A continuing challenge is the application of drug repositioning directly in the clinical practice of CLL patients.

## 6. Future Perspectives and Challenges

More accurate and reliable multilayered proteomic data are of the utmost importance to develop a CLL proteomic knowledgebase. Data from different cases and subtypes of CLLs, including data from patients or disease models with all the potential driver mutations and other adverse prognostic biomarkers data from different cellular compartments, such as mitochondria, organelles and exosomes, profiling data of PTMs such as phosphorylation, methylation and neddylation, as well as data before and after treatment with new generation therapeutic agents, could strengthen proteomics data repositories concerning CLL. The simultaneous analysis of appropriate healthy control samples for comparison is also important. More powerful and sensitive mass spectrometers that enhance the sensitivity and reproducibility of these kind of studies and at the same time detect the low abundant proteins and the transient interactions between the proteins could increase the credibility of proteomic analysis.

A further goal is the improvement of the methods for single-cell proteomics on primary cells from patients, as this method deals with the tumor heterogeneity and microenvironment interactions. This image could be completed by more comprehensive protein databases, user-friendly data processing and user-friendly drug repurposing platforms. A different approach is to switch proteomic studies from large scale ‘fishing’ experiments to more targeted approaches, that can be used in clinical practice and even in precision medicine. Hence, there is a need to establish a broad proteome panel, as well as panels of possibly deregulated cell signaling pathways and mechanisms crucial for CLL, for molecular prognostication resulting in the best treatment option. It is also important to find user-friendly tools that integrate these proteomics data with other -omics strategies in an appropriate and comprehensive manner that, through a multiparametric evaluation, will enable “the right treatment for the right person”.

All these proteomic data can contribute to the revelation of the molecular pathophysiology of CLL, covering the existing gaps, and consequently to the better diagnosis and treatment of the disease. In detail, protein prognostic biomarkers corresponding to the driver mutations and to the other prognostic biomarkers, bioenergetics, microenvironmental interactions, as well as transient protein interactions and protein interaction networks found by profiling data of PTMs, will be the next therapeutic targets of CLL.

Combined therapies targeting deregulated proteins, interaction networks and pathways, either through synergistically increasing action or through balancing the resistance mechanism, will be preferred as first line treatments versus classical anti-neoplastic agents and monotherapy. Furthermore, single-cell proteomics on primary cells from patients will enable drugs combinations that target all the heterogeneity of a tumor and eliminate MRD.

Finally, the application of MS-based proteomics on clinical practice (even for a specific proteome panel) will permit the alignment of protein prognostic biomarkers to individualized treatment options, which in turn may change depending on the disease outcome and the evolving patient’s proteomic data.

## Figures and Tables

**Table 1:** Repurposed drugs in CLL. Overview of already proposed repurposed drugs in CLL, showing the possible molecular targets (targeted proteins); the quality of scientific evidence to assess the drug repurposing evidence level (status of evidence); and the publication referring each repurposed drug (publication). The proteomics-based repurposed drugs are indicated in bold. CT: clinical trial; PhI/II: phase I/II.

Repurposed Drugs	Targeted Proteins	Status of Evidence	Publication
**acitretin**	RXRA	in silico + experimental	[56]
**alitretinoin**	RXRA	in silico	[56]
**aplidine**	MAPK8	in silico	[56]
**arsenic trioxide**	PML	in silico + CT PhI/II	[56]
auranofin	IL-1β, TNF, IL-6, thioredoxin reductase	experimental + CT PhI/II	[122]
**azacytidine**	DNA methyltransferases, DNMT1	in silico + experimental + CT PhI/II	[56,122]
belimumab	BAFF	experimental	[122]
**belinostat**	HDAC8, HDAC3	in silico	[56]
**benoxaprofen**	ALOX6	in silico	[56]
**bexarotene**	RXRA	in silico	[56]
chloroquine	autophagy-related proteins	experimental	[125]
**cladribine**	RRM2B	in silico + experimental + CT PhI/II	[56]
clemastine	sphingosine	experimental	[124]
**dasatinib**	LCK	in silico + experimental + CT PhI/II	[56]
**decitabine**	DNMT1	in silico	[56]
**diclofenac**	ALOX7	in silico	[56]
dimercaprol	N/A	experimental	[123]
**elomotecan**	TOP1	in silico	[56]
**elsamitrucin**	TOP1	in silico + CT PhII	[56]
**estramustin**	MAP2	in silico	[56]
**etretinate**	RXRA	in silico	[56]
**gossypol**	BCL2	in silico + experimental + CT PhI/II	[56]
**hydroxyurea**	RRM2B	in silico	[56]
**MK 1775**	WEE1	in silico	[56]
nelfinavir	HIV protease	experimental	[125]
**nintedanib**	LCK	in silico	[56]
**N-methyl-4-lle-cyclosporin**	PPIA	in silico	[56]
**oblimersen**	BCL2	in silico + PhI/II	[56]
**paclitaxel**	BCL2	in silico	[56]
**pazopanib**	LCK	in silico + experimental	[56]
**pentosan polysulfate**	FGF2	in silico	[56]
plicamycin	NA	experimental	[122]
podofilox	DNA topoisomerase II	experimental	[122]
**pyroxamide**	HDAC9, HDAC3	in silico	[56]
**rasagiline**	BCL2	in silico	[56]
roflumilast	PDE4	experimental	[123]
simvastatin	HMGCR, LFA-1	in silico + experimental + CT PhI	[126]
**sucralfate**	FGF4	in silico	[56]
**suradista**	FGF3	in silico	[56]
**T 0128**	TOP1	in silico	[56]
**TA 270**	ALOX5	in silico	[56]
**talmapimod**	MAPK13	in silico	[56]
**tin mesoporphyrin**	HMOX1/2	in silico	[56]
**tretinoin**	RXRA	in silico + CT PhI	[56]
**triapine**	RRM2B	in silico	[56]
**tributyrin**	HDAC7	in silico	[56]
**tributyrin**	HDAC3	in silico	[56]
**valproic acid**	ALDH5A1	in silico + experimental + CT PhI/II	[56]
**vemurafenib**	FGR	in silico	[56]

**Table 2:** Proposed repurposed drugs based on proteomics and drug repurposing data. Drug repurposing based on drug targets proposed by proteomics and drug repurposing drug targets using PanDrugs [146]. Only drugs with high scores are shown.

Stage	Drugs	Targets
**Proposed for CLL patients**	BORTEZOMIB	AIFM1, BCL2, HNRNPH1, TRAF2
	PACLITAXEL	BCL2, STMN1, TRAF2
	DOCETAXEL	BCL2, TRAF2
	ERIBULIN MESYLATE	BCL2, TRAF2
	VALPROIC ACID	BCL2, FAS, HDAC8
	VENETOCLAX	BCL2, TRAF2
	VITAMIN E	ALOX5, BCL2
**Proposed for advanced CLL patients**	ABEMACICLIB	CDK4, CDK6, CDKN2A, JUN
	ARSENIC TRIOXIDE	CD44, CDK4, CDKN2A, JUN, JUP, LEF1, MAPK8, RAP1A
	BOSUTINIB	CAV1, CDK4, CDKN2A, ITGB1, RAP1A
	MIDOSTAURIN	NPM1, RAP1A, ZAP70
	PACLITAXEL	CDKN2A, MAPK8, PDCD4
	PALBOCICLIB	CDK4, CDK6, CDKN2A, JUN
	RIBOCICLIB	CDK4, CDK6, CDKN2A, JUN
	SIROLIMUS	CCNA1, FKBP4, MAPK8, NPM1, ZAP70
	TRAMETINIB	CDKN2A, RAP1A

## Supplementary Materials

The following are available online at https://www.mdpi.com/article/10.3390/cancers13143391/s1: Figure S1. The mechanisms of action of the drugs used for CLL therapy. Figure S2. Graphical illustration of the most representative proteomic findings in CLL. Table S1. Driver mutations in 38 genes in CLL. Table S2. Drugs used for CLL therapy. Table S3a. Proteomics data in CLL. Table S3b. Proposed biomarkers by the proteomics studies in CLL and number of cases examined. Table S3c. Repurposed drugs for CLL prognosis. Table S3d. Repurposed drugs for CLL diagnosis. Table S3e. Proteomics data in CLL slim version.

## Abbreviations

MS/MS	tandem mass spectrometry
PTM	post translation modifications
M	mutated
U	unmutated
P	patient
progres.	progressive
indol.	indolent
SLL	small lymphocytic lymphoma
ALL	acute lymphoblastic leukemia
AML	acute myeloid leukemia
BL	Burkitt lymphoma
CML	chronic myeloid leukemia
DLBCL	diffuse large B cell lymphoma
HD	healthy donors
RPPA	Reverse Phase Protein Array
Cys	Cysteine

## References

[1] Mosquera Orgueira A., Antelo Rodríguez B., Díaz Arias J., González Pérez M.S., Bello López J.L. (2019). New Recurrent Structural Aberrations in the Genome of Chronic Lymphocytic Leukemia Based on Exome-Sequencing Data. Front. Genet..

[2] Rai K.R., Jain P. (2016). Chronic lymphocytic leukemia (CLL)-Then and now. Am. J. Hematol..

[3] Hallek M. (2019). Chronic lymphocytic leukemia: 2020 update on diagnosis, risk stratification and treatment. Am. J. Hematol..

[4] Zhang S., Kipps T.J. (2014). The pathogenesis of chronic lymphocytic leukemia. Annu. Rev. Pathol..

[5] Brieghel C., da Cunha-Bang C., Yde C.W., Schmidt A.Y., Kinalis S. (2020). The Number of Signaling Pathways Altered by Driver Mutations in Chronic Lymphocytic Leukemia Impacts Disease Outcome. Clin. Cancer Res..

[6] Liu Y.C., Margolskee E. (2020). Chronic lymphocytic leukemia with TP53 gene alterations: A detailed clinicopathologic analysis. Mod. Pathol..

[7] Malcikova J., Tausch E., Rossi D., Sutton L.A., Soussi T. (2018). ERIC recommendations for TP53 mutation analysis in chronic lymphocytic leukemia-update on methodological approaches and results interpretation. Leukemia.

[8] Edelmann J., Holzmann K., Tausch E., Saunderson E.A., Jebaraj B.M.C., Steinbrecher D., Dolnik A., Blätte T.J., Landau D.A., Saub J. (2020). Genomic alterations in high-risk chronic lymphocytic leukemia frequently affect cell cycle key regulators and NOTCH1-regulated transcription. Haematologica.

[9] Chiorazzi N., Rai K.R., Ferrarini M. (2005). Chronic lymphocytic leukemia. N. Engl. J. Med..

[10] Smith L.D., Minton A.R., Blunt M.D., Karydis L.I., Dutton D.A., Rogers-Broadway K.R., Dobson R., Liu R., Norster F., Hogg E. (2020). BCR signaling contributes to autophagy regulation in chronic lymphocytic leukemia. Leukemia.

[11] Rigolin G.M., Saccenti E., Guardalben E., Cavallari M., Formigaro L., Zagatti B., Visentin A., Mauro F.R. (2018). In chronic lymphocytic leukaemia with complex karyotype, major structural abnormalities identify a subset of patients with inferior outcome and distinct biological characteristics. Br. J. Haematol..

[12] Baliakas P., Jeromin S., Iskas M., Puiggros A., Plevova K., Nguyen-Khac F., Davis Z., Rigolin G.M., Visentin A., Xochelli A. (2019). Cytogenetic complexity in chronic lymphocytic leukemia: Definitions, associations, and clinical impact. Blood.

[13] Visentin A., Bonaldi L., Rigolin G.M., Mauro F.R., Martines A., Frezzato F., Imbergamo S., Scomazzon E., Pravato S., Bardi M.A. (2019). The combination of complex karyotype subtypes and IGHV mutational status identifies new prognostic and predictive groups in chronic lymphocytic leukaemia. Br. J. Cancer.

[14] Leeksma A.C., Baliakas P., Moysiadis T., Puiggros A., Plevova K., Van der Kevie-Kersemaekers A.M., Posthuma H., Rodriguez-Vicente A.E., Tran A.N., Barbany G. (2021). Genomic arrays identify high-risk chronic lymphocytic leukemia with genomic complexity: A multi-center study. Haematologica.

[15] Jondreville L., Krzisch D., Chapiro E. (2020). The complex karyotype and chronic lymphocytic leukemia: Prognostic value and diagnostic recommendations. Am. J. Hematol..

[16] Roy Chowdhury S., Banerji V. (2018). Targeting Mitochondrial Bioenergetics as a Therapeutic Strategy for Chronic Lymphocytic Leukemia. Oxid. Med. Cell. Longev..

[17] Van Attekum M.H., Eldering E., Kater A.P. (2017). Chronic lymphocytic leukemia cells are active participants in microenvironmental cross-talk. Haematologica.

[18] Zhang W., Trachootham D., Liu J., Chen G., Pelicano H., Garcia-Prieto C., Lu W., Burger J.A., Croce C.M., Plunkett W. (2012). Stromal control of cystine metabolism promotes cancer cell survival in chronic lymphocytic leukaemia. Nat. Cell Biol..

[19] Jitschin R., Braun M., Qorraj M., Saul D., Le Blanc K., Zenz T., Mougiakakos D. (2015). Stromal cell-mediated glycolytic switch in CLL cells involves Notch-c-Myc signaling. Blood.

[20] Sharif-Askari B., Doyon D., Paliouras M. (2019). Bruton’s tyrosine kinase is at the crossroads of metabolic adaptation in primary malignant human lymphocytes. Sci. Rep..

[21] Binet J.L., Auquier A., Dighiero G., Chastang C., Piguet H., Goasguen J., Vaugier G., Potron G., Colona P., Oberling F. (1981). A new prognostic classification of chronic lymphocytic leukemia derived from a multivariate survival analysis. Cancer.

[22] Hallek M., Cheson B.D., Catovsky D., Caligaris-Cappio F., Dighiero G., Döhner H., Hillmen P., Keating M., Montserrat E., Chiorazzi N. (2018). iwCLL guidelines for diagnosis, indications for treatment, response assessment, and supportive management of CLL. Blood.

[23] PDQ Supportive and Palliative Care Editorial Board (2002). Chronic Lymphocytic Leukemia Treatment (PDQ^®^): Health Professional Version. PDQ Cancer Information Summaries.

[24] Falay M., Serdar M.A., Dalgali H., Uçar M.A., Dagdaş S., Özet G. (2019). Which Markers Should the used for Diagnostic Chronic Lymphocytic Leukemia Immunophenotyping Scoring System by Flow Cytometry?. Clin. Lab..

[25] Sorigue M., Magnano L., Miljkovic M.D., Nieto-Moragas J., Santos-Gomez M., Villamor N., Junca J., Morales-Indiano C. (2020). Positive predictive value of CD200 positivity in the differential diagnosis of chronic lymphocytic leukemia. Cytom. B Clin. Cytom..

[26] Myles N., Giri P., Chim I., Kodituwakku A. (2021). The utility of CD200 expression and modified Matutes score in the diagnostic differentiation of mantle cell lymphoma and chronic lymphocytic leukemia using flow cytometry. Leuk. Lymphoma.

[27] Xie M., Huang X., Ye X., Qian W. (2019). Prognostic and clinicopathological significance of PD-1/PD-L1 expression in the tumor microenvironment and neoplastic cells for lymphoma. Int. Immunopharmacol..

[28] Mosquera Orgueira A., Antelo Rodríguez B., Díaz Arias J., Bello López J.L. (2019). Identification of new putative driver mutations and predictors of disease evolution in chronic lymphocytic leukemia. Blood Cancer J..

[29] Katsaraki K., Artemaki P.I., Papageorgiou S.G., Pappa V., Scorilas A., Kontos C.K. (2019). Identification of a novel, internal tRNA-derived RNA fragment as a new prognostic and screening biomarker in chronic lymphocytic leukemia, using an innovative quantitative real-time PCR assay. Leuk. Res..

[30] Loi E., Moi L., Fadda A., Satta G., Zucca M., Sanna S., Amini Nia S., Cabras G., Padoan M., Magnani C. (2019). Methylation alteration of SHANK1 as a predictive, diagnostic and prognostic biomarker for chronic lymphocytic leukemia. Oncotarget.

[31] Casabonne D., Benavente Y., Seifert J., Costas L., Armesto M., Arestin M., Besson C. (2020). Serum levels of hsa-miR-16-5p, hsa-miR-29a-3p, hsa-miR-150-5p, hsa-miR-155-5p and hsa-miR-223-3p and subsequent risk of chronic lymphocytic leukemia in the EPIC study. Int. J. Cancer.

[32] Del Giudice I., Raponi S., Della Starza I., De Propris M.S., Cavalli M., De Novi L.A., Cappelli L.V., Ilari C., Cafforio L., Guarini A. (2019). Minimal Residual Disease in Chronic Lymphocytic Leukemia: A New Goal?. Front. Oncol..

[33] Jain N., O’Brien S. (2016). Targeted therapies for CLL: Practical issues with the changing treatment paradigm. Blood Rev..

[34] Freeman C.L., Gribben J.G. (2016). Immunotherapy in Chronic Lymphocytic Leukaemia (CLL). Curr. Hematol. Malig. Rep..

[35] Nguyen P.H., Niesen E., Hallek M. (2019). New roles for B cell receptor associated kinases: When the B cell is not the target. Leukemia.

[36] Schiattone L., Ghia P., Scarfò L. (2019). The evolving treatment landscape of chronic lymphocytic leukemia. Curr. Opin. Oncol..

[37] Ortiz-Maldonado V., García-Morillo M., Delgado J. (2015). The biology behind PI3K inhibition in chronic lymphocytic leukaemia. Ther. Adv. Hematol..

[38] Perini G.F., Ribeiro G.N., Pinto Neto J.V., Campos L.T., Hamerschlak N. (2018). BCL-2 as therapeutic target for hematological malignancies. J. Hematol. Oncol..

[39] Sharman J., Di Paolo J. (2016). Targeting B-cell receptor signaling kinases in chronic lymphocytic leukemia: The promise of entospletinib. Ther. Adv. Hematol..

[40] Bair S.M., Porter D.L. (2019). Accelerating chimeric antigen receptor therapy in chronic lymphocytic leukemia: The development and challenges of chimeric antigen receptor T-cell therapy for chronic lymphocytic leukemia. Am. J. Hematol..

[41] Lemal R., Tournilhac O. (2019). State-of-the-art for CAR T-cell therapy for chronic lymphocytic leukemia in 2019. J. Immunother. Ther. Cancer.

[42] Forte D., Krause D.S., Andreeff M., Bonnet D., Méndez-Ferrer S. (2019). Updates on the hematologic tumor microenvironment and its therapeutic targeting. Haematologica.

[43] Xanthopoulos C., Kostareli E. (2019). Advances in Epigenetics and Epigenomics in Chronic Lymphocytic Leukemia. Curr. Genet. Med. Rep..

[44] Janovská P., Bryja V. (2017). Wnt signalling pathways in chronic lymphocytic leukaemia and B-cell lymphomas. Br. J. Pharmacol..

[45] Bhattacharya M., Sharma A.R. (2020). Interaction between miRNAs and signaling cascades of Wnt pathway in chronic lymphocytic leukemia. J. Cell. Biochem..

[46] Rosati E., Baldoni S., De Falco F., Del Papa B., Dorillo E., Rompietti C., Albi E., Falzetti F., Di Ianni M., Sportoletti P. (2018). NOTCH1 Aberrations in Chronic Lymphocytic Leukemia. Front. Oncol..

[47] Mansouri L., Wierzbinska J.A., Plass C., Rosenquist R. (2018). Epigenetic deregulation in chronic lymphocytic leukemia: Clinical and biological impact. Semin. Cancer Biol..

[48] Hao T., Li-Talley M., Buck A., Chen W. (2019). An emerging trend of rapid increase of leukemia but not all cancers in the aging population in the United States. Sci. Rep..

[49] Hallek M. (2017). Chronic lymphocytic leukemia: 2017 update on diagnosis, risk stratification, and treatment. Am. J. Hematol..

[50] Mulligan S.P., Shumack S., Guminski A. (2019). Chronic lymphocytic leukemia, skin and other second cancers. Leuk. Lymphoma.

[51] Kumar V., Ailawadhi S., Bojanini L., Mehta A., Biswas S., Sher T., Roy V., Vishnu P., Marin-Acevedo J. (2019). Trends in the risk of second primary malignancies among survivors of chronic lymphocytic leukemia. Blood Cancer J..

[52] Bond D.A., Huang Y., Fisher J.L., Ruppert A.S., Owen D.H., Bertino E.M., Rogers K.A., Bhat S.A., Grever M.R., Jaglowski S.M. (2020). Second cancer incidence in CLL patients receiving BTK inhibitors. Leukemia.

[53] Van Der Nest B.M., Leslie C., Joske D., Radeski D., White R., Cheah C.Y. (2019). Peripheral T-Cell Lymphoma Arising in Patients With Chronic Lymphocytic Leukemia. Am. J. Clin. Pathol..

[54] Enya Chen Y.C., Burgess M., Mapp S., Mollee P., Gill D., Blumenthal A., Saunders N.A. (2020). PI3K-p110δ contributes to antibody responses by macrophages in chronic lymphocytic leukemia. Leukemia.

[55] Alsagaby S.A., Alhumaydhi F.A. (2019). Proteomics insights into the pathology and prognosis of chronic lymphocytic leukemia. Saudi Med. J..

[56] Johnston H.E., Carter M.J., Larrayoz M., Clarke J., Garbis S.D., Oscier D., Strefford J.C., Steele A.J., Walewska R., Cragg M.S. (2018). Proteomics Profiling of CLL Versus Healthy B-cells Identifies Putative Therapeutic Targets and a Subtype-independent Signature of Spliceosome Dysregulation. Mol. Cell. Proteom..

[57] Thurgood L.A., Chataway T.K., Lower K.M., Kuss B.J. (2017). From genome to proteome: Looking beyond DNA and RNA in chronic lymphocytic leukemia. J. Proteom..

[58] Psatha K., Kollipara L., Voutyraki C., Divanach P., Sickmann A., Rassidakis G.Z., Drakos E., Aivaliotis M. (2017). Deciphering lymphoma pathogenesis via state-of-the-art mass spectrometry-based quantitative proteomics. J. Chromatogr. B Analyt. Technol. Biomed. Life Sci..

[59] Gupta A., Kumar A. (2014). Pros and cons of the proteomics. Biomed. J..

[60] Díez P., Góngora R., Orfao A., Fuentes M. (2017). Functional proteomic insights in B-cell chronic lymphocytic leukemia. Expert Rev. Proteom..

[61] Almaiman A.A. (2018). Proteomic Profile of Lymphoid Leukemia. J. Coll. Phys. Surg. Pak..

[62] Cochran D.A., Evans C.A., Blinco D., Burthem J., Stevenson F.K., Gaskell S.J., Whetton A.D. (2003). Proteomic analysis of chronic lymphocytic leukemia subtypes with mutated or unmutated Ig V(H) genes. Mol. Cell. Proteom..

[63] Barnidge D.R., Jelinek D.F., Muddiman D.C., Kay N.E. (2005). Quantitative protein expression analysis of CLL B cells from mutated and unmutated IgV(H) subgroups using acid-cleavable isotope-coded affinity tag reagents. J. Proteom. Res..

[64] Rees-Unwin K.S., Faragher R., Unwin R.D., Adams J., Brown P.J., Buckle A.M., Pettitt A., Hutchinson C.V., Johnson S.M., Pulford K. (2010). Ribosome-associated nucleophosmin 1: Increased expression and shuttling activity distinguishes prognostic subtypes in chronic lymphocytic leukaemia. Br. J. Haematol..

[65] Eagle G.L., Zhuang J., Jenkins R.E., Till K.J., Jithesh P.V., Lin K., Johnson G.G., Oates M., Park K., Kitteringham N.R. (2015). Total proteome analysis identifies migration defects as a major pathogenetic factor in immunoglobulin heavy chain variable region (IGHV)-unmutated chronic lymphocytic leukemia. Mol. Cell. Proteom..

[66] Thurgood L.A., Dwyer E.S., Lower K.M., Chataway T.K., Kuss B.J. (2019). Altered expression of metabolic pathways in CLL detected by unlabelled quantitative mass spectrometry analysis. Br. J. Haematol..

[67] Eagle G.L., Herbert J.M.J., Zhuang J., Oates M., Khan U.T., Kitteringham N.R., Clarke K., Park B.K., Pettitt A.R., Jenkins R.E. (2021). Assessing technical and biological variation in SWATH-MS-based proteomic analysis of chronic lymphocytic leukaemia cells. Sci. Rep..

[68] Scielzo C., Ghia P., Conti A., Bachi A., Guida G., Geuna M., Alessio M., Caligaris-Cappio F. (2005). HS1 protein is differentially expressed in chronic lymphocytic leukemia patient subsets with good or poor prognoses. J. Clin. Investig..

[69] Perrot A., Pionneau C., Nadaud S., Davi F., Leblond V., Jacob F., Merle-Béral H., Herbrecht R., Béné M.C., Gribben J.G. (2011). A unique proteomic profile on surface IgM ligation in unmutated chronic lymphocytic leukemia. Blood.

[70] Alsagaby S.A., Khanna S., Hart K.W., Pratt G., Fegan C., Pepper C., Brewis I.A., Brennan P. (2014). Proteomics-based strategies to identify proteins relevant to chronic lymphocytic leukemia. J. Proteom. Res..

[71] Glibert P., Vossaert L., Van Steendam K., Lambrecht S., Van Nieuwerburgh F., Offner F., Kipps T., Dhaenens M., Deforce D. (2014). Quantitative proteomics to characterize specific histone H2A proteolysis in chronic lymphocytic leukemia and the myeloid THP-1 cell line. Int. J. Mol. Sci..

[72] Díez P., Lorenzo S., Dégano R.M., Ibarrola N., González-González M., Nieto W., Almeida J., González M., Orfao A., Fuentes M. (2016). Multipronged functional proteomics approaches for global identification of altered cell signalling pathways in B-cell chronic lymphocytic leukaemia. Proteomics.

[73] Kashuba E., Eagle G.L., Bailey J., Evans P., Welham K.J., Allsup D., Cawkwell L. (2013). Proteomic analysis of B-cell receptor signaling in chronic lymphocytic leukaemia reveals a possible role for kininogen. J. Proteom..

[74] Díez P., Ibarrola N., Dégano R.M., Lécrevisse Q., Rodriguez-Caballero A., Criado I., Nieto W.G., Góngora R., González M., Almeida J. (2017). A systematic approach for peptide characterization of B-cell receptor in chronic lymphocytic leukemia cells. Oncotarget.

[75] Voss T., Ahorn H., Haberl P., Döhner H., Wilgenbus K. (2001). Correlation of clinical data with proteomics profiles in 24 patients with B-cell chronic lymphocytic leukemia. Int. J. Cancer.

[76] Huang P.Y., Mactier S., Armacki N., Giles Best O., Belov L., Kaufman K.L., Pascovici D., Mulligan S.P., Christopherson R.I. (2016). Protein profiles distinguish stable and progressive chronic lymphocytic leukemia. Leuk. Lymphoma.

[77] Bretones G., Álvarez M.G., Arango J.R., Rodríguez D., Nadeu F., Prado M.A., Valdés-Mas R., Puente D.A., Paulo J.A., Delgado J. (2018). Altered patterns of global protein synthesis and translational fidelity in RPS15-mutated chronic lymphocytic leukemia. Blood.

[78] Su X., Lucas D.M., Zhang L., Xu H., Zabrouskov V., Davis M.E., Knapp A.R., Young D.C., Payne P.R., Parthun M.R. (2009). Validation of an LC-MS based approach for profiling histones in chronic lymphocytic leukemia. Proteomics.

[79] Singh R., Harshman S.W., Ruppert A.S., Mortazavi A., Lucas D.M., Thomas-Ahner J.M., Clinton S.K., Byrd J.C., Freitas M.A., Parthun M.R. (2015). Proteomic profiling identifies specific histone species associated with leukemic and cancer cells. Clin. Proteom..

[80] O’Hayre M., Salanga C.L., Kipps T.J., Messmer D., Dorrestein P.C., Handel T.M. (2010). Elucidating the CXCL12/CXCR4 signaling network in chronic lymphocytic leukemia through phosphoproteomics analysis. PLoS ONE.

[81] Prieto D., Sotelo N., Seija N., Sernbo S., Abreu C., Durán R., Gil M., Sicco E., Irigoin V., Oliver C. (2017). S100-A9 protein in exosomes from chronic lymphocytic leukemia cells promotes NF-κB activity during disease progression. Blood.

[82] Haderk F., Schulz R. (2017). Tumor-derived exosomes modulate PD-L1 expression in monocytes. Sci. Immunol..

[83] Mangolini M., Götte F., Moore A. (2018). Notch2 controls non-autonomous Wnt-signalling in chronic lymphocytic leukaemia. Nat. Commun..

[84] Boyd R.S., Adam P.J., Patel S., Loader J.A., Berry J., Redpath N.T., Poyser H.R., Fletcher G.C., Burgess N.A., Stamps A.C. (2003). Proteomic analysis of the cell-surface membrane in chronic lymphocytic leukemia: Identification of two novel proteins, BCNP1 and MIG2B. Leukemia.

[85] Miguet L., Béchade G., Fornecker L., Zink E., Felden C., Gervais C., Herbrecht R., Van Dorsselaer A., Mauvieux L., Sanglier-Cianferani S. (2009). Proteomic analysis of malignant B-cell derived microparticles reveals CD148 as a potentially useful antigenic biomarker for mantle cell lymphoma diagnosis. J. Proteom. Res..

[86] Henrich S., Crossett B., Christopherson R.I. (2007). Differentially expressed nuclear proteins in human CCRF-CEM, HL-60, MEC-1 and Raji cells correlate with cellular properties. Proteom. Clin. Appl..

[87] Mayer R.L., Schwarzmeier J.D., Gerner M.C., Bileck A., Mader J.C., Meier-Menches S.M., Gerner S.M., Schmetterer K.G., Pukrop T., Reichle A. (2018). Proteomics and metabolomics identify molecular mechanisms of aging potentially predisposing for chronic lymphocytic leukemia. Mol. Cell. Proteom..

[88] Gez S., Crossett B., Christopherson R.I. (2007). Differentially expressed cytosolic proteins in human leukemia and lymphoma cell lines correlate with lineages and functions. Biochim. Biophys. Acta.

[89] Miguet L., Bogumil R., Decloquement P., Herbrecht R., Potier N., Mauvieux L., Van Dorsselaer A. (2006). Discovery and identification of potential biomarkers in a prospective study of chronic lymphoid malignancies using SELDI-TOF-MS. J. Proteom. Res..

[90] Schröder C., Srinivasan H., Sill M., Linseisen J., Fellenberg K., Becker N., Nieters A., Hoheisel J.D. (2013). Plasma protein analysis of patients with different B-cell lymphomas using high-content antibody microarrays. Proteom. Clin. Appl..

[91] Johnston H.E., Carter M.J., Cox K.L., Dunscombe M., Manousopoulou A., Townsend P.A., Garbis S.D. (2017). Integrated Cellular and Plasma Proteomics of Contrasting B-cell Cancers Reveals Common, Unique and Systemic Signatures. Mol. Cell. Proteom..

[92] Marina O., Biernacki M.A., Brusic V., Wu C.J. (2008). A concentration-dependent analysis method for high density protein microarrays. J. Proteom. Res..

[93] Khodadoust M.S., Olsson N. (2019). B-cell lymphomas present immunoglobulin neoantigens. Blood.

[94] Henrich S., Mactier S., Best G., Mulligan S.P., Crossett B., Christopherson R.I. (2011). Fludarabine nucleoside modulates nuclear “survival and death” proteins in resistant chronic lymphocytic leukemia cells. Nucleosides Nucleotides Nucleic Acids.

[95] Che Y., Best O.G., Zhong L., Kaufman K.L., Mactier S., Raftery M., Graves L.M., Mulligan S.P., Christopherson R.I. (2013). Hsp90 Inhibitor SNX-7081 dysregulates proteins involved with DNA repair and replication and the cell cycle in human chronic lymphocytic leukemia (CLL) cells. J. Proteom. Res..

[96] Kaufman K.L., Jenkins Y., Alomari M., Mirzaei M., Best O.G., Pascovici D., Mactier S., Mulligan S.P., Haynes P.A., Christopherson R.I. (2015). The Hsp90 inhibitor SNX-7081 is synergistic with fludarabine nucleoside via DNA damage and repair mechanisms in human, p53-negative chronic lymphocytic leukemia. Oncotarget.

[97] Kruse U., Pallasch C.P., Bantscheff M., Eberhard D., Frenzel L., Ghidelli S., Maier S.K., Werner T., Wendtner C.M., Drewes G. (2011). Chemoproteomics-based kinome profiling and target deconvolution of clinical multi-kinase inhibitors in primary chronic lymphocytic leukemia cells. Leukemia.

[98] Beckmann L., Berg V., Dickhut C., Sun C., Merkel O., Bloehdorn J., Robrecht S., Seifert M., da Palma Guerreiro A., Claasen J. (2021). MARCKS affects cell motility and response to BTK inhibitors in CLL. Blood.

[99] Shull A.Y., Noonepalle S.K., Awan F.T., Liu J., Pei L., Bollag R.J., Salman H., Ding Z., Shi H. (2015). RPPA-based protein profiling reveals eIF4G overexpression and 4E-BP1 serine 65 phosphorylation as molecular events that correspond with a pro-survival phenotype in chronic lymphocytic leukemia. Oncotarget.

[100] Frezzato F., Accordi B., Trimarco V., Gattazzo C., Martini V., Milani G., Bresolin S., Severin F., Visentin A., Basso G. (2016). Profiling B cell chronic lymphocytic leukemia by reverse phase protein array: Focus on apoptotic proteins. J. Leukoc. Biol..

[101] Patel V.K., Lamothe B., Ayres M.L., Gay J., Cheung J.P., Balakrishnan K., Ivan C. (2018). Pharmacodynamics and proteomic analysis of acalabrutinib therapy: Similarity of on-target effects to ibrutinib and rationale for combination therapy. Leukemia.

[102] Vangapandu H.V., Havranek O., Ayres M.L., Kaipparettu B.A., Balakrishnan K., Wierda W.G., Keating M.J., Davis R.E., Stellrecht C.M., Gandhi V. (2017). B-cell Receptor Signaling Regulates Metabolism in Chronic Lymphocytic Leukemia. Mol. Cancer Res..

[103] Langedijk J., Mantel-Teeuwisse A.K., Slijkerman D.S., Schutjens M.H. (2015). Drug repositioning and repurposing: Terminology and definitions in literature. Drug Discov. Today.

[104] Konc J. (2019). Binding site comparisons for target-centered drug discovery. Expert Opin. Drug Discov..

[105] McCabe B., Liberante F., Mills K.I. (2015). Repurposing medicinal compounds for blood cancer treatment. Ann. Hematol..

[106] Xue H., Li J., Xie H., Wang Y. (2018). Review of Drug Repositioning Approaches and Resources. Int. J. Biol. Sci..

[107] Sleire L., Førde H.E., Netland I.A., Leiss L., Skeie B.S., Enger P. (2017). Drug repurposing in cancer. Pharmacol. Res..

[108] Kaushik I., Ramachandran S., Prasad S., Srivastava S.K. (2021). Drug rechanneling: A novel paradigm for cancer treatment. Semin. Cancer Biol..

[109] Kirtonia A., Gala K., Fernandes S.G., Pandya G., Pandey A.K., Sethi G., Khattar E., Garg M. (2021). Repurposing of drugs: An attractive pharmacological strategy for cancer therapeutics. Semin. Cancer Biol..

[110] Orecchioni S., Roma S., Raimondi S., Gandini S., Bertolini F. (2019). Identifying Drug Repurposing Opportunities in Oncology. Cancer J..

[111] Armando R.G., Mengual Gómez D.L., Gomez D.E. (2020). New drugs are not enough-drug repositioning in oncology: An update. Int. J. Oncol..

[112] Olgen S., Kotra L.P. (2019). Drug Repurposing in the Development of Anticancer Agents. Curr. Med. Chem..

[113] Eriksson A., Österroos A., Hassan S., Gullbo J., Rickardson L., Jarvius M., Nygren P., Fryknäs M., Höglund M., Larsson R. (2015). Drug screen in patient cells suggests quinacrine to be repositioned for treatment of acute myeloid leukemia. Blood Cancer J..

[114] Kuenzi B.M., Remsing Rix L.L., Kinose F., Kroeger J.L., Lancet J.E., Padron E., Rix U. (2019). Off-target based drug repurposing opportunities for tivantinib in acute myeloid leukemia. Sci. Rep..

[115] Lu X., Efferth T. (2021). Repurposing of artemisinin-type drugs for the treatment of acute leukemia. Semin. Cancer Biol..

[116] Singh V.K., Chang H.H., Kuo C.C., Shiao H.Y., Hsieh H.P., Coumar M.S. (2017). Drug repurposing for chronic myeloid leukemia: In silico and in vitro investigation of DrugBank database for allosteric Bcr-Abl inhibitors. J. Biomol. Struct. Dyn..

[117] Sohraby F., Bagheri M., Aliyar M., Aryapour H. (2017). In silico drug repurposing of FDA-approved drugs to predict new inhibitors for drug resistant T315I mutant and wild-type BCR-ABL1: A virtual screening and molecular dynamics study. J. Mol. Graph. Model.

[118] Frismantas V., Dobay M.P., Rinaldi A., Tchinda J., Dunn S.H., Kunz J., Richter-Pechanska P., Marovca B., Pail O., Jenni S. (2017). Ex vivo drug response profiling detects recurrent sensitivity patterns in drug-resistant acute lymphoblastic leukemia. Blood.

[119] Scuoppo C., Wang J., Persaud M., Mittan S.K., Basso K., Pasqualucci L., Rabadan R., Inghirami G., Grandori C., Bosch F. (2019). Repurposing dasatinib for diffuse large B cell lymphoma. Proc. Natl. Acad. Sci. USA.

[120] Karube K., Enjuanes A., Dlouhy I., Jares P., Martin-Garcia D., Nadeu F. (2018). Integrating genomic alterations in diffuse large B-cell lymphoma identifies new relevant pathways and potential therapeutic targets. Leukemia.

[121] Han C., Yu X., Zhang C., Cai Y., Cao Y., Wang S., Shen J. (2019). Drug Repurposing Screen Identifies Novel Classes of Drugs with Anticancer Activity in Mantle Cell Lymphoma. Comb. Chem. High Throughput Screen.

[122] Shen M., Zhang Y., Saba N., Austin C.P., Wiestner A., Auld D.S. (2013). Identification of therapeutic candidates for chronic lymphocytic leukemia from a library of approved drugs. PLoS ONE.

[123] Cooney J.D., Lin A.P., Jiang D., Wang L., Suhasini A.N., Myers J., Qiu Z., Wölfler A., Sill H., Aguiar R.C.T. (2018). Synergistic Targeting of the Regulatory and Catalytic Subunits of PI3Kδ in Mature B-cell Malignancies. Clin. Cancer Res..

[124] Chanas-Larue A., Villalpando-Rodriguez G.E., Henson E.S., Johnston J.B., Gibson S.B. (2020). Antihistamines are synergistic with Bruton’s tyrosine kinase inhibiter ibrutinib mediated by lysosome disruption in chronic lymphocytic leukemia (CLL) cells. Leuk. Res..

[125] Mahoney E., Maddocks K., Flynn J., Jones J., Cole S.L., Zhang X., Byrd J.C., Johnson A.J. (2013). Identification of endoplasmic reticulum stress-inducing agents by antagonizing autophagy: A new potential strategy for identification of anti-cancer therapeutics in B-cell malignancies. Leuk. Lymphoma.

[126] Gimenez N., Tripathi R., Giro A., Rosich L., Lopez-Guerra M., Lopez-Oreja I., Playa-Albinyana H., Arenas F., Mas J.M., Perez-Galan P. (2020). Systems biology drug screening identifies statins as enhancers of current therapies in chronic lymphocytic leukemia. Sci. Rep..

[127] Pushpakom S., Iorio F., Eyers P.A., Escott K.J., Hopper S., Wells A., Doig A., Guilliams T., Latimer J., McNamee C. (2019). Drug repurposing: Progress, challenges and recommendations. Nat. Rev. Drug Discov..

[128] Lotfi Shahreza M., Ghadiri N., Mousavi S.R., Varshosaz J., Green J.R. (2018). A review of network-based approaches to drug repositioning. Brief Bioinform..

[129] Gns H.S., Gr S., Murahari M., Krishnamurthy M. (2019). An update on Drug Repurposing: Re-written saga of the drug’s fate. Biomed. Pharmacother..

[130] Glicksberg B.S., Li L., Chen R., Dudley J., Chen B. (2019). Leveraging Big Data to Transform Drug Discovery. Methods Mol. Biol..

[131] Cheng F., Desai R.J. (2018). Network-based approach to prediction and population-based validation of in silico drug repurposing. Nat. Commun..

[132] Ozdemir E.S., Halakou F., Nussinov R., Gursoy A., Keskin O. (2019). Methods for Discovering and Targeting Druggable Protein-Protein Interfaces and Their Application to Repurposing. Methods Mol. Biol..

[133] Banovic P., Stankov S., Vranjes N., Zurkovic O., Capo I., Lalosevic D. (2018). Drug repurposing: Mebendazole as effective antitumor agent. Are we seeing the whole story?. J. Buon..

[134] Cavalla D. (2019). Using human experience to identify drug repurposing opportunities: Theory and practice. Br. J. Clin. Pharmacol..

[135] Pulley J.M., Rhoads J.P., Jerome R.N., Challa A.P., Erreger K.B., Joly M.M., Lavieri R.R., Perry K.E., Zaleski N.M., Shirey-Rice J.K. (2020). Using What We Already Have: Uncovering New Drug Repurposing Strategies in Existing Omics Data. Annu. Rev. Pharmacol. Toxicol..

[136] Chiu Y.C., Chen H.H., Gorthi A., Mostavi M., Zheng S., Huang Y., Chen Y. (2020). Deep learning of pharmacogenomics resources: Moving towards precision oncology. Brief Bioinform..

[137] Qian T., Zhu S., Hoshida Y. (2019). Use of big data in drug development for precision medicine: An update. Expert Rev. Precis. Med. Drug Dev..

[138] Yoshida G.J. (2020). Regulation of heterogeneous cancer-associated fibroblasts: The molecular pathology of activated signaling pathways. J. Exp. Clin. Cancer Res..

[139] Laganà A., Beno I., Melnekoff D., Leshchenko V., Madduri D., Ramdas D., Sanchez L., Niglio S., Perumal D., Kidd B.A. (2018). Precision Medicine for Relapsed Multiple Myeloma on the Basis of an Integrative Multiomics Approach. JCO Precis. Oncol..

[140] Zhu F.X., He Y.C., Zhang J.Y., Wang H.F., Zhong C., Wang X.T. (2019). Using Prognosis-Related Gene Expression Signature and Connectivity Map for Personalized Drug Repositioning in Multiple Myeloma. Med. Sci. Monit..

[141] Conte F., Fiscon G., Licursi V., Bizzarri D., D’Antò T., Farina L., Paci P. (2020). A paradigm shift in medicine: A comprehensive review of network-based approaches. Biochim. Biophys. Acta Gene Regul. Mech..

[142] Nicora G., Vitali F., Dagliati A., Geifman N., Bellazzi R. (2020). Integrated Multi-Omics Analyses in Oncology: A Review of Machine Learning Methods and Tools. Front. Oncol..

[143] Tanoli Z., Alam Z., Ianevski A., Wennerberg K., Vähä-Koskela M., Aittokallio T. (2018). Interactive visual analysis of drug-target interaction networks using Drug Target Profiler, with applications to precision medicine and drug repurposing. Brief Bioinform..

[144] Gorshkov K., Chen C.Z., Marshall R.E., Mihatov N., Choi Y., Nguyen D.T., Southall N., Chen K.G., Park J.K., Zheng W. (2019). Advancing precision medicine with personalized drug screening. Drug Discov. Today.

[145] Velez G., Bassuk A.G., Colgan D., Tsang S.H., Mahajan V.B. (2017). Therapeutic drug repositioning using personalized proteomics of liquid biopsies. JCI Insight.

[146] Pineiro-Yanez E., Reboiro-Jato M., Gomez-Lopez G., Perales-Paton J., Troule K., Rodriguez J.M., Tejero H., Shimamura T., Lopez-Casas P.P., Carretero J. (2018). PanDrugs: A novel method to prioritize anticancer drug treatments according to individual genomic data. Genome Med..

[147] Seetharaman S., Etienne-Manneville S. (2020). Cytoskeletal Crosstalk in Cell Migration. Trends Cell Biol..

