# Supplementary material for: Proteomics and Drug Repurposing in CLL towards Precision Medicine
Source: Cancers (Basel). 2021 Jul 6;13(14):3391. doi: 10.3390/cancers13143391 (PMC8303629; doi:10.3390/cancers13143391)
Supplement: Supplementary file 1 [file cancers-13-03391-s001.zip › Supplementary Materials.pdf]

Supplementary Materials

The following are available online:

Figure S1. The mechanisms of action of the drugs used for CLL therapy. The most representative categories of these drugs are (i) chemotherapeutics (alkylating agents and nucleotides) that act on the nuclear of the cells, (ii) BAKs inhibitors (PI3K, BTK, LYN and SYK inhibitors) targeting BCR signaling, (iii) BCL-2 inhibitors targeting the apoptotic pathway through mitochondria and (iv) monoclonal antibodies (against CD20 or CD52), (v) checkpoints inhibitors and (vi) CAR-T therapy, that act on the cancerous microenvironment.

Figure S2. Graphical illustration of the most representative proteomic findings in CLL. Studies include among others IGHV mutational status, BCR signaling, cytogenetics and driver mutations, cancerous microenvironment, prognostic biomarkers and comparative analysis with normal cells, other diseases and pharmaceutical treatments. Many of these data belong in more than one type. [● : UM-CLL, ● : types of proteins, ● : healthy donors, ● : treatment, ● : cytogenetic aberrations, ● : genetic mutations, ● : other diseases].

Table S1. Driver mutations in 38 genes in CLL. CLL is characterized by highly varying genetic mutations, with just a handful of mutated genes common to more than 5% of patients. All these mutations are particularly useful as prognostic biomarkers of the disease, as well as putative indicators for therapeutic treatment options (+: many indications found, ++: clinical practice, -: no indications found yet).

Table S2. Drugs used for CLL therapy. Representative categories of CLL drugs: chemotherapeutics (nitrogen mustards, antimetabolites) and monoclonal antibodies, and BAKs- and BCL-2 inhibitors.

Table S3a. Proteomics data in CLL. Overview of the proteomics studies in CLL (table S3a represents an extended version of "Table 3"). Each column depicts the main characteristics of the studies presented. SAMPLES and COMPARISON: The type of biological model used in each study; METHOD: The method used for the proteomic analysis; OUTCOME: The main findings of each study. Biomarkers depicted in red are "reverse" biomarkers, which means that they found downregulated and may have a protective role against the disease or disease progression.

Table S3b. Proposed biomarkers by the proteomics studies in CLL and number of cases examined. Biomarkers are divorced on DIAGNOSTIC BIOMARKERS that could distinguish CLL patients from healthy/ patients of other diseases; and PROGNOSTIC BIOMARKERS that could predict the clinical outcome of CLL patients.

Table S3c. Repurposed drugs for CLL prognosis. Proposed prognostic biomarkers were aligned to drugs presented in the platform DrugBank using the drug repurposing tool bioDBnet (biological DataBase network).

Table S3d. Repurposed drugs for CLL diagnosis. Proposed diagnostic biomarkers were aligned to drugs presented in the platform DrugBank using the drug repurposing tool bioDBnet (biological DataBase network).

Table S3e. Proteomic studies in CLL slim version. The most representative studies involve IGHV mutational status, BCR signaling, cytogenetics and driver mutations, histones, cancerous microenvironments, prognostic biomarkers, subcellular compartments (nucleus, plasma membranes, lipid rafts, cytosols), comparison with normal cells or other diseases, and pharmaceutical treatments. With bold letters are indicated the studies that belong to more than one category. MS/MS: tandem mass spectrometry; PTM: post translation modification; RPPA: reverse-phase protein array.

Abbreviations: MS/MS: tandem mass spectrometry; PTM: post translation modifications; M: mutated; U: unmutated; P: patient; progres.: progressive; indol.: indolent; SLL: small lymphocytic lymphoma; ALL: acute lymphoblastic leukemia; AML: acute myeloid leukemia; BL: Burkitt lymphoma; CML: chronic myeloid leukemia; DLBCL: diffuse large B-cell lymphoma; HD: healthy donors; RPPA: Reverse Phase Protein Array; Cys: Cysteine
