# Supplementary figures and images for: Proteomics and Drug Repurposing in CLL towards Precision Medicine
Source: Cancers (Basel). 2021 Jul 6;13(14):3391. doi: 10.3390/cancers13143391 (PMC8303629; doi:10.3390/cancers13143391)

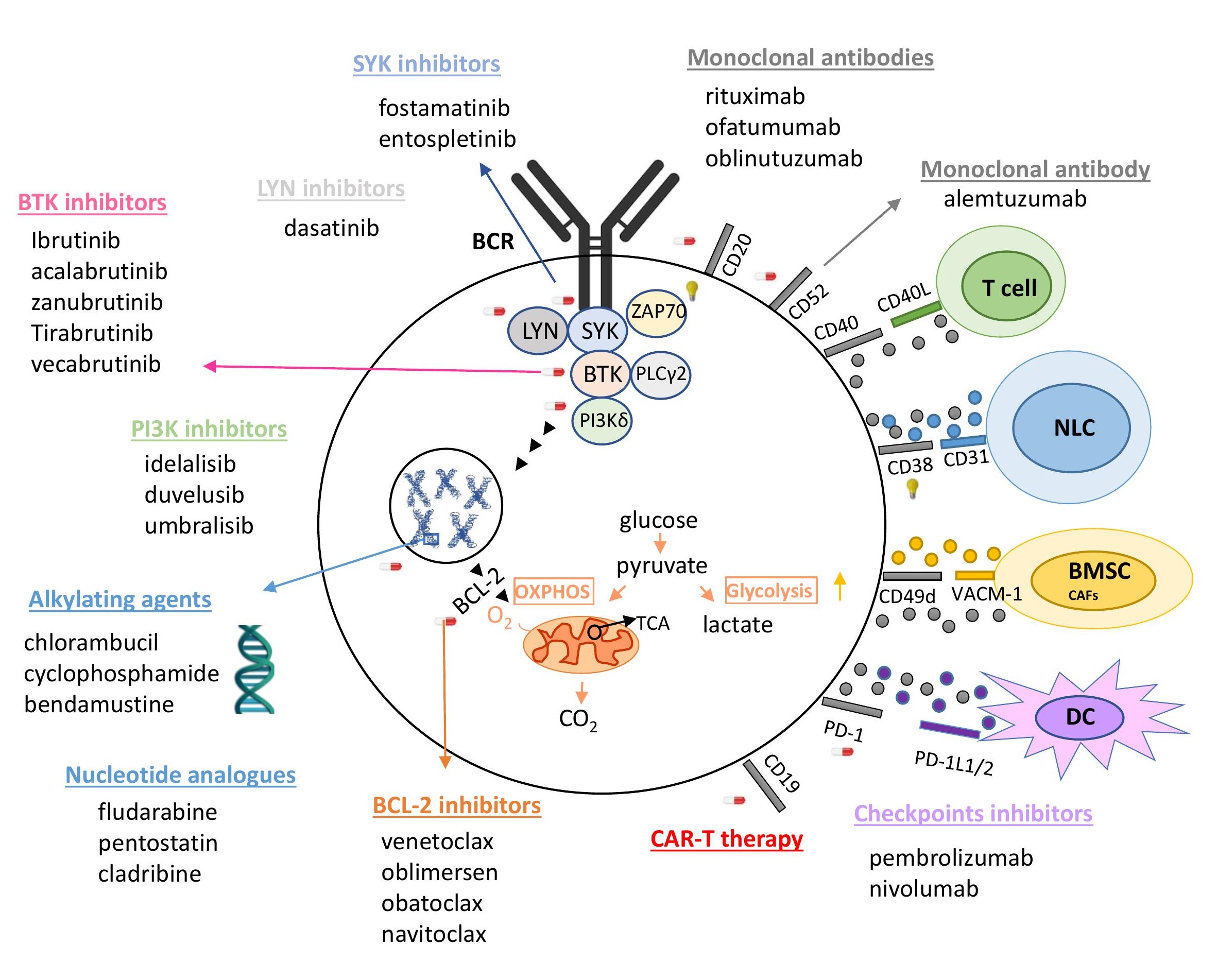

Supplement: Supplementary file 1 [file cancers-13-03391-s001.zip › Figure S1 drugs in CLL.jpg]

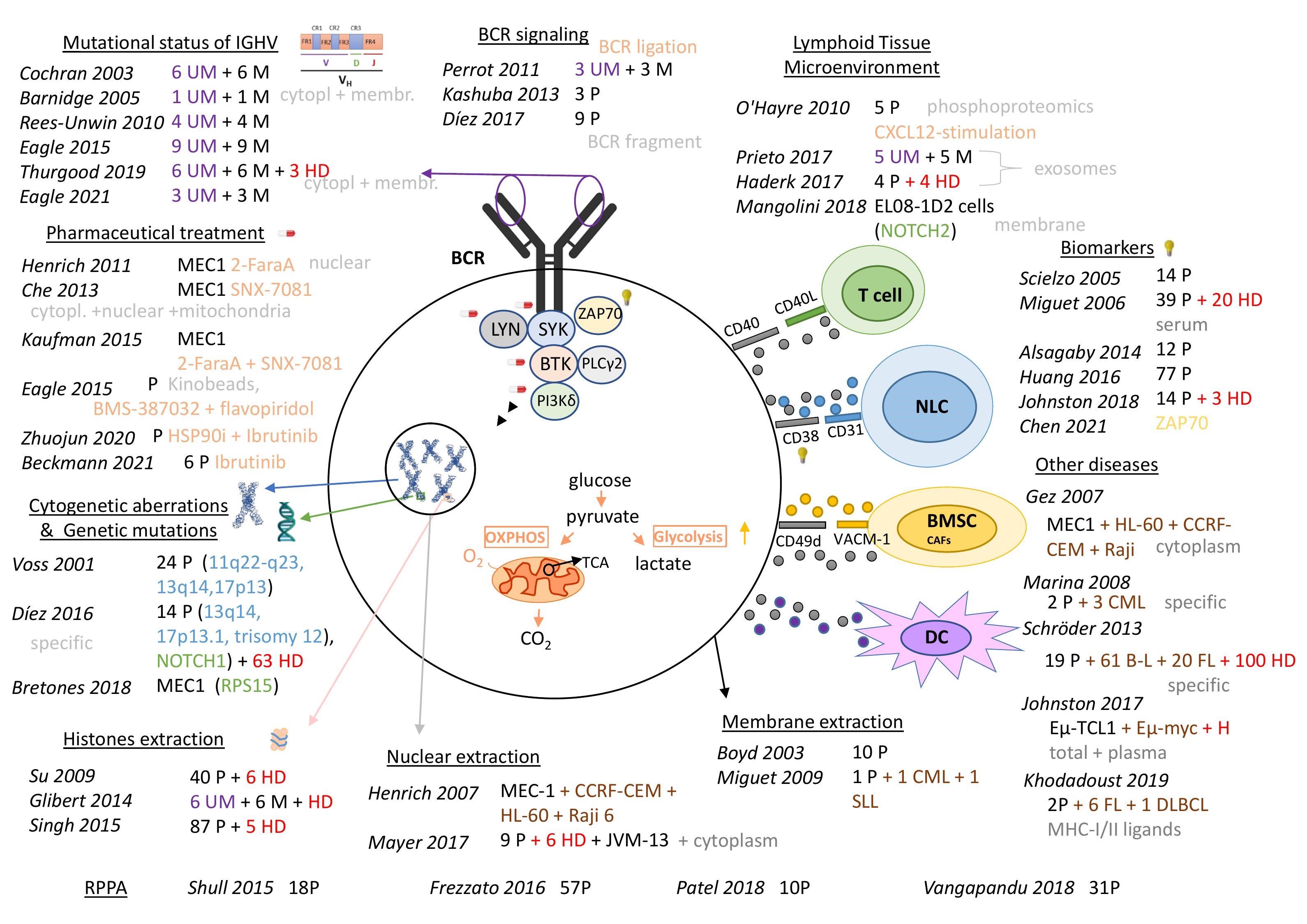

Supplement: Supplementary file 1 [file cancers-13-03391-s001.zip › Figure S2 proteomics in CLL.jpg]
